# Characterization of mRNA-Cytoskeleton Interactions *In Situ* Using FMTRIP and Proximity Ligation

**DOI:** 10.1371/journal.pone.0074598

**Published:** 2013-09-11

**Authors:** Jeenah Jung, Aaron W. Lifland, Eric J. Alonas, Chiara Zurla, Philip J. Santangelo

**Affiliations:** Wallace H. Coulter Department of Biomedical Engineering, Georgia Institute of Technology and Emory University, Atlanta, Georgia, United States of America; McGill University, Canada

## Abstract

Many studies have demonstrated an association between the cytoskeleton and mRNA, as well as the asymmetric distribution of mRNA granules within the cell in response to various signaling events. It is likely that the extensive cytoskeletal network directs mRNA transport and localization, with different cytoskeletal elements having their own specific roles. In order to understand the spatiotemporal changes in the interactions between the mRNA and the cytoskeleton as a response to a stimulus, a technique that can visualize and quantify these changes across a population of cells while capturing cell-to-cell variations is required. Here, we demonstrate a method for imaging and quantifying mRNA-cytoskeleton interactions on a per cell basis with single-interaction sensitivity. Using a proximity ligation assay with flag-tagged multiply-labeled tetravalent RNA imaging probes (FMTRIP), we quantified interactions between mRNAs and β-tubulin, vimentin, or filamentous actin (F-actin) for two different mRNAs, poly(A) + and β-actin mRNA, in two different cell types, A549 cells and human dermal fibroblasts (HDF). We found that the mRNAs interacted predominantly with F-actin (>50% in HDF, >20% in A549 cells), compared to β-tubulin (<5%) and vimentin (11-13%). This likely reflects differences in mRNA management by the two cell types. We then quantified changes in these interactions in response to two perturbations, F-actin depolymerization and arsenite-induced oxidative stress, both of which alter either the cytoskeleton itself and mRNA localization. Both perturbations led to a decrease in poly(A) + mRNA interactions with F-actin and an increase in the interactions with microtubules, in a time dependent manner.

## Introduction

mRNA localization regulates gene expression spatially and temporally by directing transcripts to restricted subcellular compartments for translation and degradation at the appropriate time [1,2]. For example, during stress, mRNAs are degraded in localized foci, such as processing bodies and RNA exosomes [3]. β-actin mRNAs in fibroblasts are localized to the leading edge where actin polymerization promotes forward protrusions [4-6]. Multiple labs have observed an association between mRNA and the cytoskeleton [7-10]. Using electron microscopy (EM), poly(A)+ mRNA in human fibroblasts has been found in association with actin filaments mostly and with vimentin and microfilaments less frequently [11]. These interactions between mRNA (generally, the 3′UTR), motor proteins, and cytoskeleton have been shown to drive the localization of mRNAs in various model systems, such as yeast [12,13], Drosophila oogenesis [14,15], and neurons [16,17]. In both human dermal fibroblasts (HDF) and A549 cells, an epithelial cell line, β-actin mRNAs exhibit processive, active transport along the microtubules using kinesin and dynein, while they likely are anchored on the actin filaments [18], facilitating translation [6,19].

Many experiments for detecting mRNA-cytoskeleton interactions and identifying the *cis*- and trans-acting factors [20,21] have been performed using biochemical assays, such as electrophoretic mobility shift [22] and immunoprecipitation [23,24], and imaging methods, such as *in situ* hybridization [11,25]. For RNA detection, methods often use exogenously expressed RNAs with MS2, which may be imaged [26,27], isolated, and analyzed for binding factors [28]. While these tools are useful in identifying RNA-binding proteins (RBP) and their binding sites, which mediate mRNA transport, a new technology that is both high-throughput and amenable to the study of native mRNAs and proteins, such cytoskeletal proteins, at their native expression levels, is needed [2]. Although the *in situ* hybridization EM data [11] conclusively described the frequency of interactions between native poly(A)+ mRNA and cytoskeletal elements, this approach is low-throughput, laborious, and expensive. It also may introduce sampling errors due to the evaluation of thin sections. The ability to image and quantify these interactions with single interaction sensitivity on a per cell basis will allow us to better understand the significance of mRNA-cytoskeleton interactions in mRNA expression and function.

In this report, using flag-tagged multiply-labeled tetravalent RNA imaging probes (FMTRIP), native mRNA can be detected in live cells without requiring transgenic manipulation [18,29,30]. Combining these probes with proximity ligation and rolling circle amplification (RCA) [31,32], we used single-interaction sensitive proximity ligation assay (PLA) to detect poly(A) + [30] and β-actin mRNA [18] interactions with β-tubulin, vimentin, and filamentous-actin (F-actin), in HDF and A549 cells. β-actin mRNA localization has been studied widely in a variety of cell types, such as fibroblasts, epithelial cells, and neurons, which makes it a suitable model for validating this method [6].

Here, we demonstrate that PLA can serve as a useful tool for imaging and quantifying mRNA-cytoskeleton interactions. For the first time, mRNA-cytoskeletal interactions for different mRNAs were compared quantitatively. PLA accurately detected interactions between mRNA and the cytoskeleton rather than simply imply that they are in proximity to each other [31]. Consistent with previous EM findings [11], mRNAs were bound predominantly to actin, compared to microtubules and vimentin, in both A549 and HDF. Actin filaments likely maintain mRNAs in a stationary state, while microtubules guide moving mRNAs. Depolymerization of the cytoskeleton reduces interactions between mRNA and cytoskeleton, and disrupts mRNA localization [33,34], even though mRNA is colocalized with the cytoskeleton.

We perturbed the cytoskeleton via cytochalasin D (cytoD) and sodium arsenite, and characterized the resulting changes in the mRNA-cytoskeleton interactions. CytoD simply depolymerized actin, while sodium arsenite, in addition to affecting mRNA localization and interactions with the cytoskeletal elements, also induces oxidative stress and alters the translational potential. CytoD-induced F-actin depolymerization and the resulting disruption of mRNA-F-actin interactions increased interactions between mRNA and β-tubulin and decreased interactions between mRNA and vimentin. During arsenite-induced oxidative stress, we also observed an increase in the mRNA binding with microtubules associated with a decrease in their binding with F-actin.

## Materials and Methods

### FMTRIP synthesis

Flag-tagged neutravidin was synthesized by first conjugating flag tag-hyNic (Solulink) to neutravidin (Thermo) modified with 4FB (Solulink) using the manufacturer’s protocol. The concentration of flag tag-hyNic and 4FB-modified neutravidin were adjusted to produce a molar ratio of two flags per neutravidin. After flag labeling, FMTRIPs were assembled as previously described [29]. Briefly, 2′-O-methyl RNA-DNA oligonucleotide chimeras were designed with a 5′-biotin and dT-C6-NH2 internal modifications (Biosearch Technologies). Cy3B-NHS ester fluorophores (GE Healthcare) were conjugated to the oligonucleotide amine groups using the manufacturer’s protocol. Free dye was removed using 3 kDa Amicon spin columns (Millipore). The purified, labeled oligonucleotides were then tetramerized by incubation for 1 h at RT with flag-tagged neutravidin at molar ratio of 5:1. Free ligands were removed using 30 kDa Amicon spin columns (Millipore). The FMTRIP targeting different mRNA sequences (Table S12) were assembled separately prior to delivery. Neutravidin lacking the flag tag was used to assemble MTRIPs for a negative control.

### Cell lines

Primary human dermal fibroblasts (Lonza, Basel, Switzerland) and A549 lung carcinoma cells (ATCC CCL-185) were maintained in High Glucose DMEM (Lonza) with 10% FBS (Hyclone), 100 U/ml penicillin (Invitrogen), and 100 µg/ml streptomycin (Invitrogen). Cells were plated on No. 1.5 glass coverslips (Ted Pella) one day prior to infection, transfection or imaging.

### Probe delivery

For probe delivery, cells were washed in Dulbecco’s Phosphate Buffered Saline (DPBS) without Ca^2+^ and Mg^2+^ (Lonza), and then incubated with 0.2 U/ml activated streptolysin O (Sigma) in OptiMEM (Invitrogen) containing FMTRIP (60 nM poly(A) or 10 nM each of 5 β-actin-targeting probes) for 10 min at 37°C. Delivery media were replaced with growth media for 15 min to restore membrane integrity before fixation.

### Proximity ligation assay

After probe delivery and recovery, cells were fixed with either methanol or 1% paraformaldehyde (Electron Microscopy Science) in PBS, unless noted otherwise, for 10 min; permeabilized using acetone for 2 min (post-methanol fixation) or 0.2% triton X-100 (Sigma) for 5 min (post-PFA fixation); and blocked for 45 min with a modified blocking solution, which consists of 0.5% Tween 20 (CalBioChem), 0.1% Triton X-100, 0.1% gelatin (Aurion), 2% donkey serum (VWR) and 1% bovine serum albumin (BSA) (EMD) in PBS. Cells were washed with PBS for 5 min. Then they were incubated for 30 min at 37°C in each of two primary antibodies (Ab) diluted in 0.25% gelatin, 0.5% Triton X-100, 0.5% donkey serum and 1% BSA in PBS, and then corresponding oligonucleotide-labeled PLA probes (Olink Bioscience) diluted in 0.05% Tween-20 in PBS. They were washed with Duolink wash solution (Olink Bioscience) after each Ab incubation. The ligation and RCA reactions (Olink Bioscience) were performed as instructed in the manufacturer’s protocol. Then the cells were immunostained or DAPI-stained (Invitrogen) and mounted on slides using Prolong (Invitrogen).

### Antibodies

Primary Ab were mouse monoclonal anti-flag (1:500 for IF, 1:1000 for PLA, Sigma), rabbit polyclonal anti-flag (1:500 for IF, 1:1000 for PLA, Sigma), mouse monoclonal anti-β-tubulin (1:100 for IF, 1:1000 for PLA, Developmental Studies Hybridoma Bank), rabbit polyclonal anti-α-tubulin (1:200 for IF, 1:1500 for PLA, Abcam), mouse monoclonal anti-vimentin (1:50 for IF, 1:1000 for PLA, Developmental Studies Hybridoma Bank), rabbit anti-alexa fluor 488 (1:100 for IF, 1:2000 for PLA, Molecular Probes). Rabbit polyclonal anti-flag Ab was used with mouse anti-β-tubulin and mouse anti-vimentin Ab. Mouse monoclonal anti-flag Ab was used with rabbit anti-alexa fluor 488 Ab. Alexa fluor 488 Phalloidin (Molecular Probes) was used to stain actin fibers, which were then targeted with rabbit anti-alexa fluor 488 Ab for PLA. Goat polyclonal anti-nucleolin antibody (1:200 for IF, 1:500 for PLA, Santa Cruz) and mouse monoclonal anti-histone H1 antibody (1:200 for IF, 1:500 for PLA, Santa Cruz) were used as negative controls.

### Drugs

After probe delivery, cells were incubated for 90 min at 37°C in glucose free DMEM (Invitrogen) containing 1µM cytochalasin D (Sigma) for actin depolymerization, for 90 min with 4µM nocodazole (Sigma) for microtubule depolymerization, and for 4 hr with 6mM acrylamide (Sigma) for vimentin depolymerization [35]. For oxidative stress, cells were incubated for 5, 10, 20, and 40 min at 37°C in 0.5mM sodium arsenite. The cells were fixed after incubation.

### Fluorescence imaging

Unless specified otherwise, all the images were taken using a laser scanning confocal microscope, Zeiss LSM 700 using a 63×, NA 1.4 Plan-Apochromat objective. Resolution was set to 1036 × 1036. Files were imported into Volocity and linearly contrast enhanced for display. Widefield images were taken on an Axiovert 200M microscope (Zeiss) with a 63× numerical aperture (NA) 1.4 Plan-Apochromat objective, and an ORCA-ER AG camera (Hamamatsu). The imaging was performed using the Volocity acquisition software (PerkinElmer). Image stacks were recorded at 200 nm intervals to adequately sample volumes for iterative deconvolution.

### Image processing and analysis

Widefield images were deconvolved using Volocity’s deconvolution algorithms. Probe and PLA signal quantification were computed in Volocity and imported into Excel (Microsoft) or Sigma Plot (Systat) for further analysis and plotting. Images presented have been linearly contrast enhanced for clarity. All calculations were performed directly on raw, deconvolved widefield data.

### Image quantification

The volume of RNA, volume of PLA-colocalized RNA, and PLA frequency/RNA volume were measured using Volocity. Each cell was analyzed individually as follows. Each cell was identified by cytoskeletal immunostaining. The RNA volume was determined based on the SD intensity. The PLA signal initially was identified as PLA objects by their SD intensity then separated into individual punctae using the “separate touching objects” tool. The objects were further filtered based on size and maximum intensity. The RNA volume colocalized with PLA signal was determined selecting the RNA volume with PLA fluorescence intensity greater than the minimum intensity or one SD intensity below the average intensity of the PLA objects detected in the cell, whichever value was greater. For each experiment, we analyzed at least 30 representative cells; experiments were repeated at least twice. In Sigma Plot, Kruskal-Wallis one-way ANOVA was used to compare the mean probe volume, the mean percentage of probe colocalized with PLA, and the mean PLA frequency. Multiple pairwise comparisons were performed with Dunn’s method.

## Results

### Detecting interactions between mRNA and the cytoskeleton using FMTRIP and PLA

**Figure 1:**
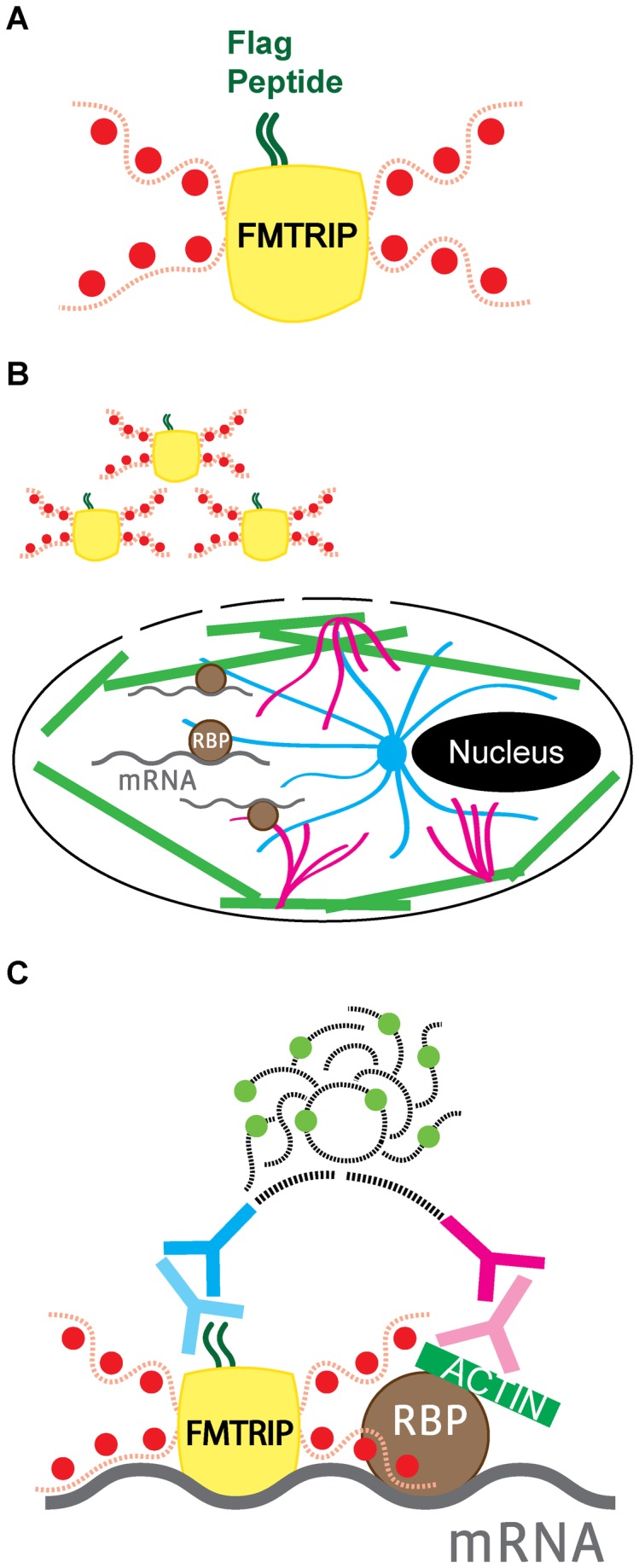
Detection of interactions between FMTRIP-hybridized mRNA and cytoskeletal elements using proximity ligation assay (PLA). (**A**) Flag (dark green) bound to a neutravidin (yellow) with Cy3B-conjugated (red) oligonucleotides (red dash) formed an FMTRIP. (**B**) Streptolysin O created entrance for FMTRIP in the cell membrane, allowing access to the mRNA (gray) bound to the β-tubulin (blue), vimentin (magenta), and F-actin (green), via RNA-binding proteins (RBP, brown). (**C**) Antibodies against the flag (light blue) and the cytoskeletal element (light magenta) in addition to the proximity probes against the antibodies (dark blue and magenta) attached to the FMTRIP-bound mRNA (gray) and the cytoskeleton (green); the oligonucleotides (black dash) on the proximity probes join to synthesize a Cy5-equivalent hybridized DNA product (light green and black dash) via rolling circle amplification.

Our general rational is that by employing FMTRIP probes and PLA, mRNA interactions with the cytoskeleton can be detected and quantified. In order to achieve this, Cy3B-labeled FMTRIP targeting poly(A) + or β-actin mRNA (Figure 1A) were delivered into live cells using streptolysin O (SLO) (Figure 1B), and post-hybridization, the cells were fixed [31]. Using proximity ligation [31,32], interactions between the flag peptide on FMTRIP-hybridized mRNA and the cytoskeletal elements, β-tubulin, vimentin, or F-actin, were detected in the fixed cells (Figure 1C). Each interaction between the FMTRIP and the cytoskeleton produced a PLA product, a Cy5-equivalent labeled DNA punctae, which can easily be identified and counted. Thus, the interactions were quantified in all image planes (Figure S1) as both the percentage of FMTRIP volume colocalized with PLA and the frequency of PLA punctae per FMTRIP volume.

Since the mRNA-cytoskeleton interactions were observed after fixation, we examined the effect of various fixatives. Specifically, we compared paraformaldehyde (PFA) diluted in PBS or in BRB80 (a tubulin retaining fixative) and methanol. We found significant differences between PFA and methanol. Methanol fixation provided the best immunofluorescence (IF) images and maintained the mRNA interactions with microtubules (Figure S2) and vimentin (Figure S3), while no difference was observed in vimentin IF images (Figure S3A). We observed a significant difference in the percentage of FMTRIP colocalized with PLA and the frequency of PLA punctae (Figure S3C, D), even though no difference was observed in the volume of FMTRIP imaging probes (Figure S3B). For phalloidin, PFA fixation proved to be optimal, confirming protocols provided by Molecular Probes (Invitrogen, Inc.). Hence, fixatives may alter mRNA-cytoskeleton interactions. Delivering and hybridizing RNA probes in live cells allowed for a variety of fixatives to be used and obviated the need for antigenicity-reducing formamide, a reagent typically used in FISH assays. Conventional FISH assays, where hybridization to RNA occurs post-fixation, typically use PFA, which may alter mRNA-protein interactions. Therefore, using this general methodology, which allows for flexibility of fixative usage, mRNA-cytoskeleton interactions can be detected and quantified accurately.

### Depolymerization of the Cytoskeletal Elements Disrupts Their Interactions with the mRNA

**Figure 2:**
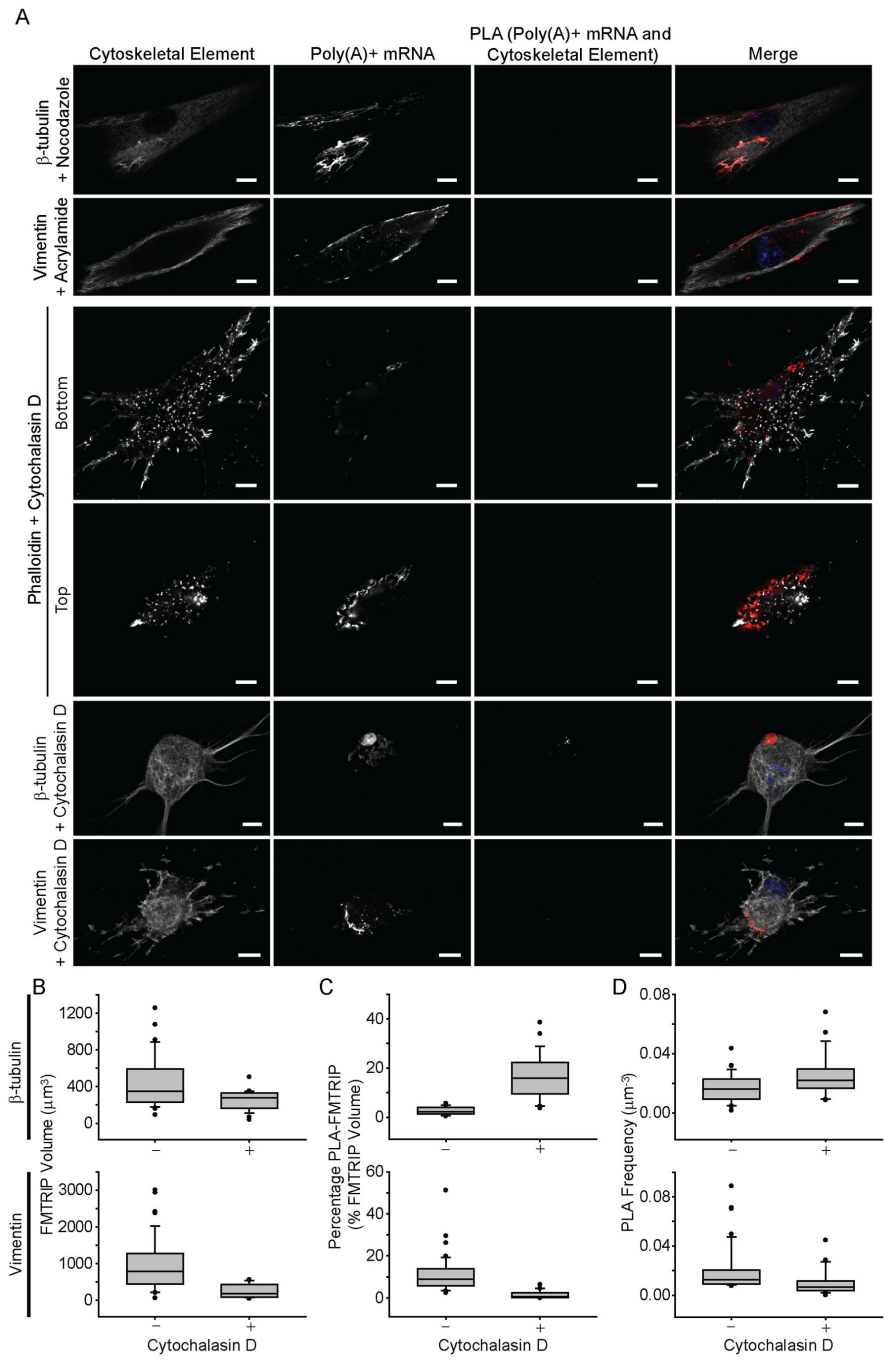
Interactions between poly(A)+ mRNA and cytoskeletal elements in HDF post-depolymerization of microtubules using nocodazole, intermediate filaments using acrylamide, and actin using cytochalasin D. (**A**) β-tubulin, vimentin, and phalloidin immunofluorescence (IF), poly(A)+ mRNA, and PLA between poly(A)+ mRNA and the cytoskeletal elements in HDF were imaged with a laser-scanning confocal microscope. Merged images of the cytoskeleton (white), poly(A)+ mRNA (red), PLA (green) and nuclei (blue) are shown. Single image plane is represented. Scale bar, 10 µm. (**B**) The mean FMTRIP volume decreased after 90 min exposure to cytochalasin D (Mann-Whitney rank sum test, β-tubulin, p<0.01; vimentin, p<0.01) in experiments quantifying interactions with β-tubulin (n=21, mean=254µm^3^, s.d.=110µm^3^) and vimentin (n=16, mean=253µm^3^, s.d.=188µm^3^). (**C**) The mean percentage of FMTRIP (PLA-FMTRIP) bound to β-tubulin increased after depolymerization (Mann-Whitney rank sum test, p<0.001; n=21, mean=16.3%, s.d.=9.5%) but decreased for vimentin (Mann-Whitney rank sum test, p<0.001; n=16, mean=1.5%, s.d.=1.9%). (**D**) The mean PLA frequency detecting interactions with β-tubulin also increased (Mann-Whitney rank sum test, p=0.015; n=21, mean=0.03µm^-3^, s.d.=0.02µm^-3^) and decreased for vimentin (Mann-Whitney rank sum test, p<0.001; n=16, mean=0.01µm^-3^, s.d.=0.01µm^-3^). Error bars, s.d.

Next, the effect of cytoskeletal depolymerization was interrogated. These experiments act as a generalized control for this methodology and confirmation of the detection of interactions between the mRNA and cytoskeleton rather than mere proximity. poly(A)-targeting FMTRIP were delivered into cells, and PLA was performed between poly(A)+ mRNA and the cytoskeletal elements, after depolymerizing microtubules with nocodazole, vimentin with acrylamide, and actin with cytochalasin D (cytoD). Depolymerization in HDF (Figure 2) and in A549 cells (Figure S4) resulted in no PLA signal, although the mRNA appeared near the depolymerized elements.

Depolymerizing β-tubulin didn’t appear to affect the distribution of the mRNA, as they remained widely dispersed throughout the cell (Figure 2, Figure S4). Disrupting vimentin and actin led to observable differences in the localization of the mRNA. In vimentin-depolymerized cells, more mRNA clustered around the nucleus, while mRNA in the actin-depolymerized cells moved toward the nucleus as well as the top of the cell. The vertical distribution of the mRNA was more apparent especially in the HDFs (Figure 2).

### Poly(A)+ mRNAs are bound predominantly to actin in HDF and A549 cells

**Figure 3:**
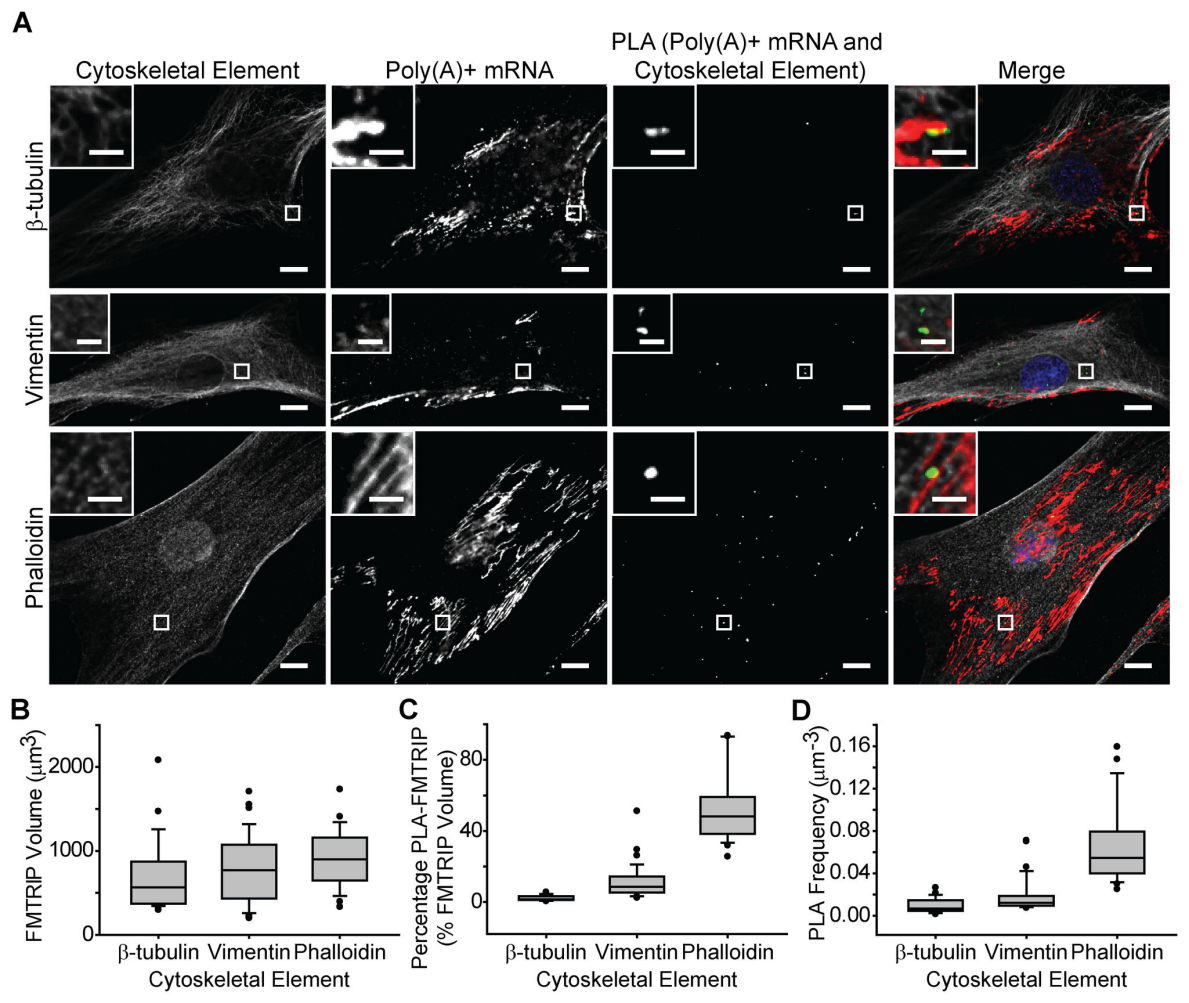
Interactions between poly(A)+ mRNA and cytoskeletal elements in HDF. (**A**) β-tubulin, vimentin and phalloidin IF, poly(A)+ mRNA, and PLA between poly(A)+ mRNA and the cytoskeletal elements in HDF were imaged with a laser-scanning confocal microscope. Merged images of the cytoskeleton (white), poly(A)+ mRNA (red), PLA (green) and nuclei (blue) are shown. Single image plane is represented. Inset, images of boxed regions. Scale bar, 10 µm (2 µm in insets). (**B**) The mean FMTRIP volume was similar (Kruskal-Wallis One Way ANOVA on Ranks, p=0.5) in cells, where the interactions between poly(A)+ mRNA and β-tubulin (n=25, mean=706µm^3^, s.d.=427µm^3^), vimentin (n=33, mean=780µm^3^, s.d.=405µm^3^), or phalloidin (n=23, mean=916µm^3^, s.d.=346µm^3^) were quantified. (**C**) The mean percentage of FMTRIP colocalized with PLA (PLA-FMTRIP) was significantly different (Table S1) between the interactions of poly(A)+ mRNA with β-tubulin (n=25, mean=2.3%, s.d.=1.5%), vimentin (n=33, mean=11.5%, s.d.=9.7%), or phalloidin (n=23, mean=53.7%, s.d.=19.9%). (**D**) The mean PLA frequency was significantly different (Table S2) between the interactions of poly(A)+ mRNA with β-tubulin (n=25, mean=0.010µm^-3^, s.d.=0.007µm^-3^), vimentin (n=33, mean=0.019µm^-3^, s.d.=0.016µm^-3^), or phalloidin (n=23, mean=0.069µm^-3^, s.d.=0.039µm^-3^). Error bars, s.d.

Following the control experiments, a general characterization of poly(A)+ mRNA was performed in HDF and A549 cells. Poly(A)-targeting FMTRIPs were delivered to HDF and A549 cells at 60nM concentration, which is less than the concentration that resulted in the maximum intensity (90nM). By under-sampling, we labeled a random portion of the mRNA population, which facilitated imaging individual mRNA granules and quantifying relative differences in their interactions with the cytoskeleton. After hybridization and fixation, the interactions of poly(A)+ mRNA with β-tubulin, vimentin or actin (marked by phalloidin) were imaged (Figure 3A, Figure S5A) and quantified (Figure 3B-D, Figure S5B-D). The fibroblasts generally were larger and more spread out than the epithelial cells. However, the FMTRIP signal was dispersed throughout the cytoplasm of both cell types; the mean FMTRIP volume was greater in the HDF than in the A549 cells (Figure 3A, Figure S5A). We observed no difference in the FMTRIP delivery and hybridization between the experimental groups by the volume of the granules (Figure 3B, Figure S5B).

**Figure 4:**
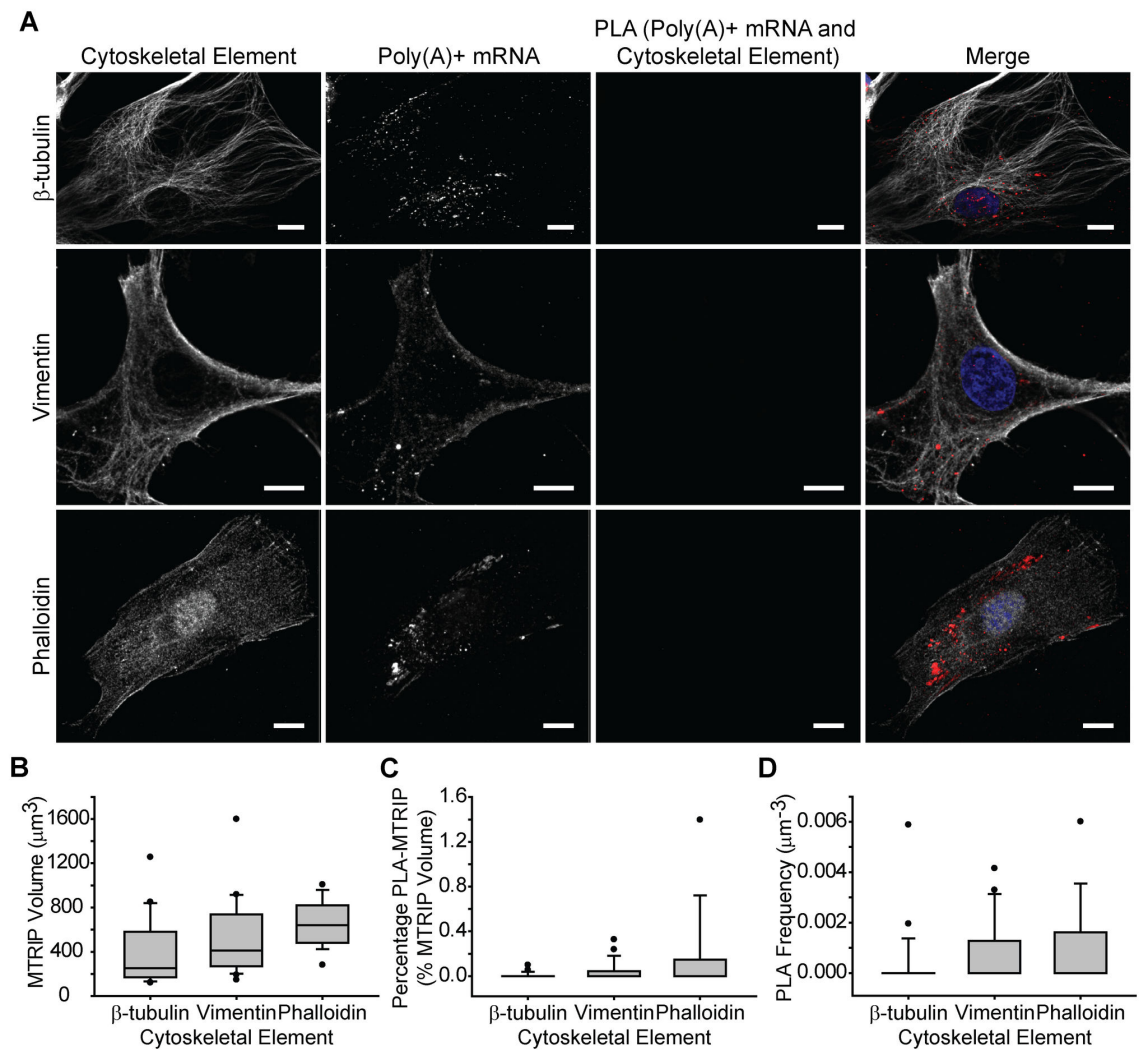
Interactions between poly(A)+ mRNA bound to MTRIP lacking flag tag and cytoskeletal elements in human dermal fibroblasts (HDF). (**A**) β-tubulin, vimentin and phalloidin IF, poly(A)+ mRNA, and PLA between poly(A)+ mRNA, and the cytoskeletal elements in HDF were imaged with a laser-scanning confocal microscope. Merged images of the cytoskeleton (white), poly(A)+ mRNA (red), PLA (green), and nuclei (blue) are shown. Single image plane is represented. Scale bar, 10 µm. (**B**) The mean MTRIP volume was similar (Kruskal-Wallis One Way ANOVA on Ranks, p=0.08) in cells, where the interactions between β-actin mRNA and β-tubulin (n=18, mean=404µm^3^, s.d.=317µm^3^), vimentin (n=19, mean=518µm^3^, s.d.=362µm^3^), or phalloidin (n=15, mean=653µm^3^, s.d.=211µm^3^) were quantified. (**C**) The mean percentage of MTRIP colocalized with PLA (PLA-MTRIP) was similarly minimal (Kruskal-Wallis One Way ANOVA on Ranks, p=0.15) in β-tubulin (n=18, mean=0.01%, s.d.=0.03%), vimentin (n=19, mean=0.04%, s.d.=0.1%), or phalloidin (n=15, mean=0.20%, s.d.=0.41%). (**D**) The mean PLA frequency was also minimal (Kruskal-Wallis One Way ANOVA on Ranks, p=0.23) in β-tubulin (n=18, mean=0.0004µm^-3^, s.d.=0.001µm^-3^), vimentin (n=19, mean=0.0008µm^-3^, s.d.=0.0001µm^-3^), or phalloidin (n=15, mean=0.001µm^-3^, s.d.=0.002µm^-3^). Error bars, s.d.

Contrastingly, the mean percentage of FMTRIP volume colocalized with PLA signal (PLA-FMTRIP) as well as the mean PLA frequency significantly differed between mRNA interactions with various cytoskeletal elements. Although the poly(A)+ mRNA and β-tubulin IF appeared colocalized, their interactions were less than 5% for both HDF and A549 cells. In the HDF, on average, 2.3% FMTRIP volume colocalized with PLA signal between the mRNA and β-tubulin (Figure 3C); this was consistent in A549 cells (Figure S5C). Consistently, the mean PLA frequency normalized by the cell’s mRNA volume also was minimal for the HDF (Figure 4D) and the A549 cells (Figure S5D). The minimal percentage of mRNA bound to β-tubulin was due to the minimal number of interactions as detected by PLA, rather than smaller aggregates of mRNA bound to β-tubulin. Interactions between the mRNA and vimentin were more frequent in comparison. The mean percentage of PLA-FMTRIP was 11.5% in the HDF (Figure 3C); this was similar in the A549 (n=28) (Figure S5C). The mean PLA frequency also was greater for the HDF (n=33) (Figure 3D) and the A549 cells (Figure S5D).

As previously observed by Bassell et al using EM [11], we also observed that in HDF, significantly more poly(A)+ mRNA, 53.7%, was bound to the F-actin (Figure 3C). The mean PLA frequency was consistently the greatest (Figure 3D). However, for A549, there was no significant difference (p>0.05) between vimentin and F-actin (Figure S5C,D and Table S4). In the A549 cells, less mRNA colocalized with PLA between the mRNA and F-actin compared to the HDFs (Figure S5C). The mean PLA frequency also was less (Figure S5D). Generally, the PLA signal was localized to the branching points of actin filaments (Figure 3A inset, Figure S5A inset), similarly to the EM findings [11].

As an additional negative control, MTRIPs lacking the flag tag were delivered to HDF (Figure 4) and A549 cells (Figure S6). The delivery and hybridization for the MTRIPs were similar to the FMTRIPs in HDFs (Figure 4B) and A549 cells (Figure S6B). Only a few interactions, likely due to non-specific interactions, were observed between the mRNA and cytoskeletal elements. In HDFs, the mean percentage of PLA-MTRIP was minimal (<0.2%) (Figure 4C). The mean PLA frequency also was insignificant (Figure 4D). Consistently, in A549 cells, the mean percentage of PLA-MTRIP was <0.05% (Figure S6C). The mean PLA frequency was insignificant (Figure S6D). The data clearly show that poly(A)+ mRNA was predominately bound to actin.

### β-actin mRNAs also are bound predominantly to actin

**Figure 5:**
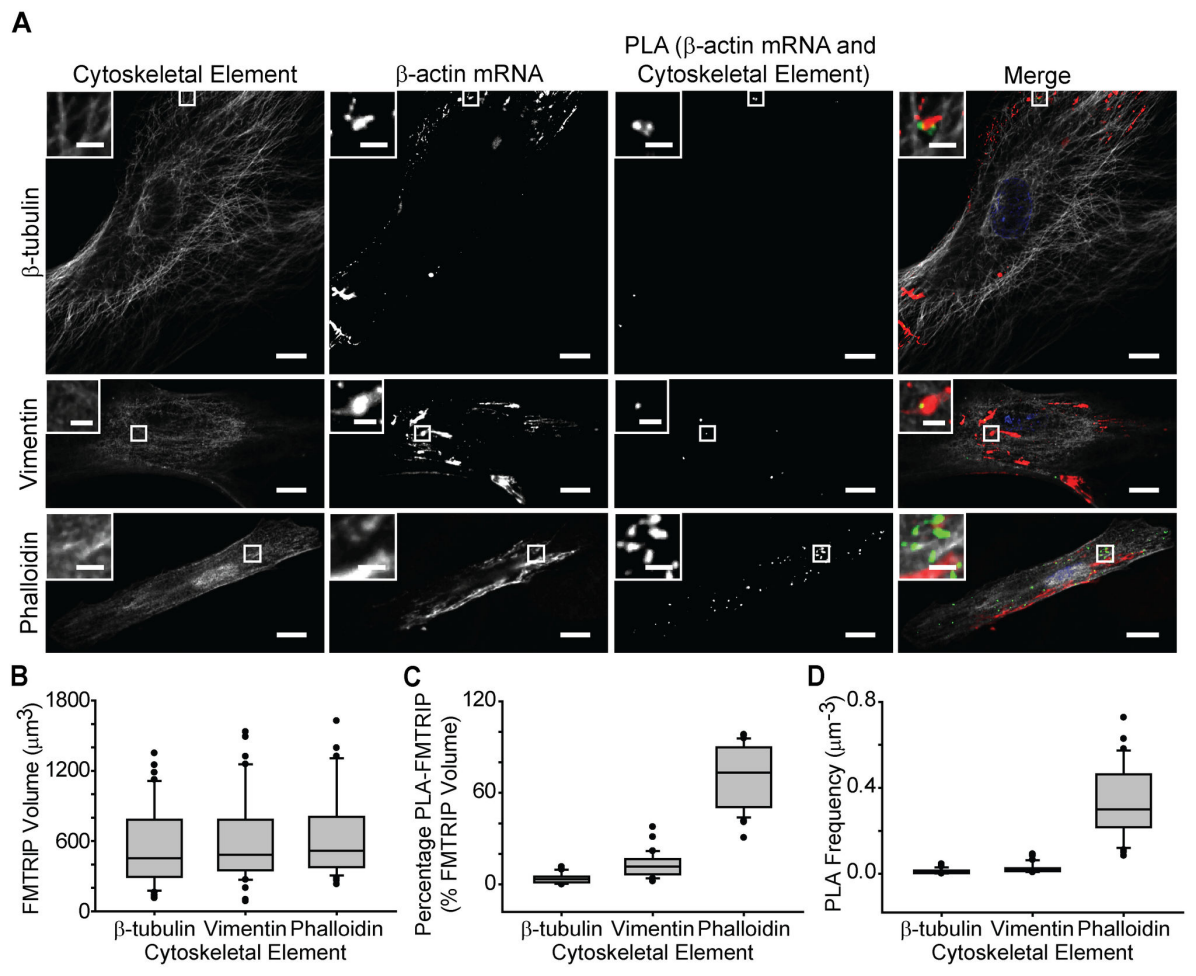
Interactions between β-actin mRNA and cytoskeletal elements in HDF. (**A**) β-tubulin, vimentin and phalloidin IF, β-actin mRNA, and PLA between β-actin mRNA and the cytoskeletal elements in HDF were imaged with a laser-scanning confocal microscope. Merged images of the cytoskeleton (white), β-actin mRNA (red), PLA (green), and nuclei (blue) are shown. Single image plane is represented. Inset, images of boxed regions. Scale bar, 10 µm (2 µm in insets). (**B**) The mean FMTRIP volume was similar (Kruskal-Wallis One Way ANOVA on Ranks, p=0.5) in cells, where the interactions between β-actin mRNA and β-tubulin (n=40, mean=567µm^3^, s.d.=355µm^3^), vimentin (n=36, mean=607µm^3^, s.d.=385µm^3^), or phalloidin (n=30, mean=660µm^3^, s.d.=389µm^3^) were quantified. (**C**) The mean percentage of FMTRIP colocalized with PLA (PLA-FMTRIP) was significantly different (Table S5) between the interactions of β-actin mRNA with β-tubulin (n=40, mean=3.9%, s.d.=3.2%), vimentin (n=36, mean=12.7%, s.d.=7.9%), or phalloidin (n=30, mean=71.5%, s.d.=20.1%). (**D**) The mean PLA frequency was significantly different (Table S6) between the interactions of β-actin mRNA with β-tubulin (n=40, mean=0.012µm^-3^, s.d.=0.011µm^-3^), vimentin (n=36, mean=0.027µm^-3^, s.d.=0.023µm^-3^), or phalloidin (n=30, mean=0.33µm^-3^, s.d.=0.17µm^-3^). Error bars, s.d.

In order to evaluate the localization of a specific mRNA, we delivered 50nM FMTRIP targeting the untranslated and translated regions of β-actin mRNA [31]. As previously observed [18,29], FMTRIP-bound β-actin mRNA was prevalent in the perinuclear region and the leading edges of the cell (Figure 5A, Figure S7A). The range of FMTRIP volume detected in cells was similar for the HDFs and A549 cells. No difference in the delivery and binding of FMTRIP was observed between the experimental groups (Figure 5B, Figure S7B). Similar to poly(A)+ mRNAs, in both HDF and A549 cells, majority of β-actin mRNAs interacted with F-actin than the other cytoskeletal elements (Figure 5, Figure S7). In HDFs, on average, 3.9% of β-actin FMTRIP interacted with β-tubulin, 12.7% with vimentin, and 71.5% with F-actin (Figure 5C). The mean frequency of PLA was consistent with the percentage of FMTRIP (Figure 5D). In A549 cells, 2.6% β-actin FMTRIP interacted with β-tubulin, 11.8% with vimentin, and 31.3% with F-actin (Figure S7C). The mean PLA frequency again was consistent (Figure S7D).

β-actin MTRIP lacking the flag tag resulted in minimal PLA signal (Figure S8, S9). No difference in the probe delivery or hybridization was observed between β-actin FMTRIP and MTRIP for the HDF (Figure S8B) and for the A549 cells (Figure S9B). Minimal interaction was observed between the β-actin mRNA and the cytoskeletal elements in HDFs (Figure S8B-D) and A549 cells (Figure S9B-D). As negative controls, we used PLA to detect β-actin mRNA interactions with nuclear proteins, nucleolin and histone H1. Since the nuclear proteins are predominately within the nucleus, they generally do not interact with the cytoplasmic β-actin mRNA. FMTRIPs only target the cytoplasmic mRNAs; therefore, we detected no PLA signal between β-actin mRNAs and the nuclear proteins (Figure S10). As with poly(A)+ mRNA, β-actin mRNA were predominately bound to filamentous actin.

### Depolymerization of F-actin leads to increased mRNA binding to the microtubules

In our initial experiments, depolymerization was utilized as a control experiment, but this process also could be used as general perturbation of the cytoskeleton. Once we determined that depolymerization altered mRNA interactions with the depolymerized element, we asked whether this altered mRNA interactions with the other, intact, cytoskeletal elements. When F-actin is depolymerized, the cell morphology was noticeably affected. The cytoplasm was reduced significantly with retracted edges, and the mRNA localized in the perinuclear region (Figure 2A). Generally, the detected mRNA volume also decreased (Figure 2B). However, while the interactions with F-actin were eliminated, the mean percentage of poly(A) + mRNA interacting with β-tubulin increased, while interactions with vimentin decreased (Figure 2C). Consistently, the mean PLA frequency for interactions with β-tubulin increased, while interactions with vimentin decreased (Figure 2D). Hence, changes in the percentage of mRNA interacting with β-tubulin or vimentin were due to changes in the number of PLA, rather than larger mRNA granules colocalizing with PLA. Considering the dramatic changes in the cell morphology, F-actin depolymerization might have also disrupted other cytoskeletal components, such as microtubules and intermediate filaments. However, since poly(A) + mRNA interactions with β-tubulin and vimentin resulted in PLA products, β-tubulin and vimentin likely were not disrupted significantly by cytoD, since their depolymerization would have decreased the PLA signal to undetectable levels (Figure 2A).

The increase in poly(A)+ mRNA binding to β-tubulin and decrease in the binding to vimentin suggest that as mRNA granules separate from F-actin, due to its depolymerization, and likely bind to microtubules. Microtubules have been observed to transport mRNA granules [18,30], while F-actin has been thought to anchor and translate the mRNA. Moving mRNA granules are unlikely to undergo translation; hence, once the mRNAs separate from F-actin and vimentin, they may bind to microtubules to be transported elsewhere. This will be explored in future experiments. From these experiments, it is clear that depolymerization of F-actin leads to altered mRNA localization, specifically to the microtubules.

### Arsenite-induced oxidative stress decreased mRNA binding to F-actin and increased binding to microtubules

**Figure 6:**
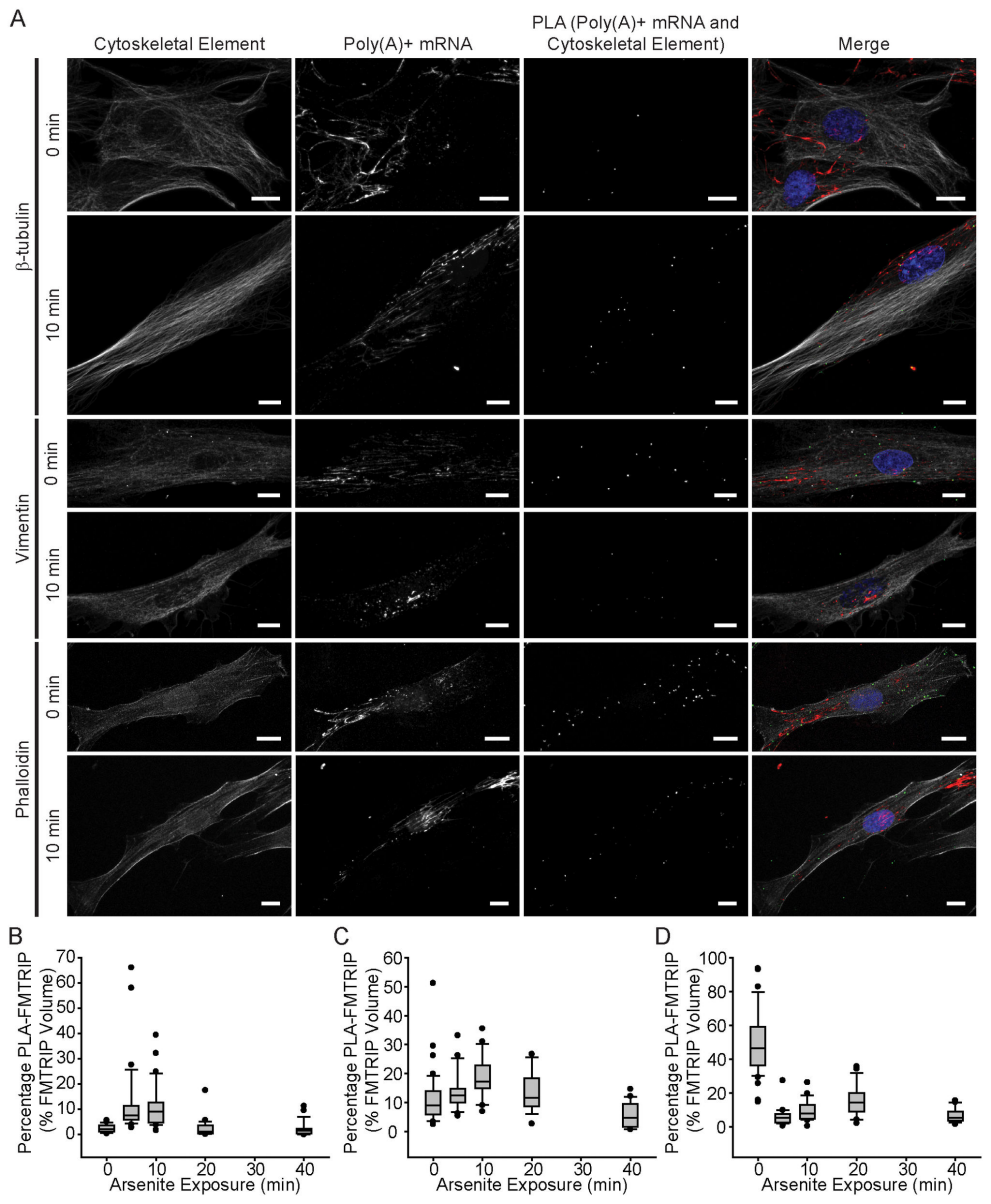
Arsenite-induced oxidative stress reduced poly(A)+ mRNA binding to F-actin and vimentin, while increasing interactions with β-tubulin. (A) β-tubulin, vimentin and phalloidin IF, poly(A)+ mRNA, and PLA between poly(A)+ mRNA and the cytoskeletal elements in HDF were imaged at 0 min (or no) and 10 min exposure to arsenite with a laser-scanning confocal microscope. Merged images of the cytoskeleton (white), poly(A)+ mRNA (red), PLA (green), and nuclei (blue) are shown. All image planes are represented. Scale bar, 10 µm. (B) The mean percentage of FMTRIP (PLA-FMTRIP) bound to β-tubulin increased significantly from 0 min exposure (n=28, mean=2.5%, s.d.=1.6%) to 5 min exposure (Kruskal-Wallis One Way ANOVA on Ranks, p<0.001; n=31, mean=12.8%,s.d.=14.6%); remained high for 10 min exposure (n=26, mean=11.3%, s.d.=9.1%); and decreased to the pre-exposure level at 20 (Kruskal-Wallis One Way ANOVA on Ranks, p<0.05; n=22, mean=2.6%, s.d.=3.8%) and 40 min (n=20, mean=2.2%, s.d.=3.0%) (Table S9). (C) The mean percentage of FMTRIP (PLA-FMTRIP) bound to vimentin remained similar to 0 min exposure (n=42, mean=11.1%, s.d.=8.8%) for 5 (n=23, mean=13.9%,s.d.=6.5%),10 (n=19, mean=18.7%, s.d.=7.5%), and 20 min (n=13, mean=13.3%, s.d.=7.1%) exposure; it decreased at 40 min (n=17, mean=5.9%, s.d.=4.4%) (Table S10). (D) The mean percentage of FMTRIP (PLA-FMTRIP) bound to F-actin decreased significantly from 0 min exposure (n=38, mean=49.3%, s.d.=19.4%) to 5 min exposure (Kruskal-Wallis One Way ANOVA on Ranks, p<0.001; n=25, mean=6.0%,s.d.=5.2%); remained low for 10 (n=21, mean=9.6%, s.d.=6.4%), 20 (n=19, mean=15.4%, s.d.=9.4%), and 40 min (n=16, mean=6.8%, s.d.=4.4%) exposure (Table S11). Error bars, s.d.

In order to further investigate the relationship between mRNA binding to various cytoskeletal components, we examined interactions between poly(A)+ mRNA and the cytoskeletal elements at various time points during arsenite-induced oxidative stress. Arsenite is known to induce stress granule formation and alter mRNA localization and translational potential; therefore, a relevant perturbation to the cell. After 5-10 min of arsenite exposure, the poly(A) + mRNA interactions with β-tubulin increased significantly. Interactions significantly decreased at 20 min and remained minimal until 40 min. (Figure 6A, B, S11, Table S9) Contrastingly, interactions with F-actin decreased significantly at 5 min and remained low throughout the experiment (Figure 6A, D, S13, Table S10). Changes in the interactions with vimentin were not as dramatic. Generally, they remained similar until 40 min, when the percentage mRNAs interacting with vimentin reached their minimum (Figure 6A, C, S12, Table S11).

Zurla et al (2011) observed that 15 min after arsenite exposure, the β-actin mRNA granules began to accumulate near the nucleus and microtubule-organizing center (MTOC) [30]. After 30 min, the cells’ morphology changed dramatically with their edges retracted toward the nucleus. They also found that depolymerizing microtubules using nocodazole prevented β-actin mRNA from localizing to the MTOC [30]. The increase in the poly(A) + mRNA interactions with β-tubulin and the decrease in the interactions with F-actin within the first 10 min of exposure are consistent with previous findings. mRNA granules anchored to F-actin likely separate and bind to microtubules for transport to the perinuclear region and MTOC. Zurla et al found that within 10 min, the mRNA granules at the cell periphery were not recruited toward the MTOC possibly because they remained bound to the cytoskeleton [30]. At 10 min after exposure, we also observed PLA signals around the periphery (Figure 6A), which eventually disappeared with extended exposure. By 40 minutes, interactions with β-tubulin, vimentin, and F-actin all decreased to minimal levels. This was likely due to arsenite-induced cytoskeleton instability. Exposure to arsenite led to cell retraction and loss of actin filaments; at higher doses, loss of microtubules and intermediate filaments was observed as well as inhibition of cytoskeletal protein synthesis [36,37]. In these experiments, we found that arsenite alters mRNA localization with the cytoskeleton, promoting interactions with the microtubules over F-actin.

## Discussion

In contrast to the 48 hours [38] required to image and quantify interactions between poly(A)+ mRNA and cytoskeleton in five to ten cells using EM [11], using the combined FMTRIP and PLA method, this was achievable for two different mRNAs in 15-40 cells within eight hours using a widefield fluorescent microscope. Since single interactions lead to the production of single fluorescent puncta [32], mRNA-cytoskeleton interactions were easily identified and quantified on a per cell basis. With the mRNAs also hybridized to fluorescent probes that reflect their quantity [30,31], the interactions were normalized by the mRNA signal, allowing quantification that can be compared between cells. Because probes were hybridized to the mRNA in live cells before fixation, they targeted mRNAs that were undistorted by fixatives. The ability to detect these interactions quantitatively using multiple fixatives post-probe delivery, allowed us to utilize the optimal fixative for each cytoskeletal element, ensuring accuracy; *this was not possible with other methods*.

In addition to these methodological advantages, our findings were consistent with the EM data [11], both poly(A) + and β-actin mRNA were bound predominantly to actin, compared to microtubules and vimentin, in both A549 cells and HDF. In human diploid fibroblasts, Bassell et al. observed 72% poly(A)+ mRNA was localized within 5nm of F-actin filaments (n=5); 29% with vimentin filaments (n=5); and less than 10% with microtubules (n=5) [11]. In human dermal fibroblasts, on average, we observed 54% poly(A) + mRNA interacting with F-actin (n=23); 12% with vimentin (n=33); and 2% with β-tubulin (n=25). For β-actin mRNA, 72% interacted with F-actin (n=30), 13% with vimentin (n=36), and 4% with β-tubulin (n=40). The lower mean percentage of poly(A) + mRNA interaction using PLA compared to EM is likely because we under-labeled mRNA. The maximum intensity was obtained when 90nM probes were delivered [18,29]; 60nM concentrations of probes were delivered in order to label a random portion of the mRNA population and to facilitate the imaging of individual mRNA granules and quantification of relative differences in their interactions with the cytoskeleton. In addition, the PLA reaction depends on the antibodies bound to the cytoskeleton and FMTRIP. Antibody concentrations that were less than that for maximal coverage were utilized, in order to sample a random portion of the protein population and to limit the detection of non-specific interactions. This is acceptable because the main purpose of using PLA for detection mRNA-protein interactions is to quantify and compare the relative change in the interactions, rather than to determine the absolute number of interactions. Slight differences between the EM data and our findings might be due to our larger sample size, methodological differences in data acquisition and analysis, and differences in the cell types used.

This work supports the idea that mRNAs are bound predominantly to actin, compared to microtubules and vimentin. mRNAs are likely anchored to actin [6] and transported to specific areas in the cell by microtubules associated with motors [18]. Similar to actin, vimentin likely serves to anchor the mRNA rather than to direct its motion. Vimentin depolymerization dramatically changed the mRNA localization for both mRNAs in both cell types, similar to actin depolymerization. Contrastingly, microtubule depolymerization had little effect on the mRNA distribution.

On average, a greater percentage of mRNA was bound to F-actin in the HDFs compared to A549 cells, even though the percentage of mRNA bound to β-tubulin and vimentin was similar in both cell types. During epithelial to mesenchymal transition (EMT) in various cell types, translation of actin and vimentin increases along with actin rearrangement [39]. One hypothesis may be that since A549 cells are an adenocarcinoma cell line and likely exhibit increased mesenchymal characteristics than normal epithelial cells, such as greater production of vimentin and actin [40,41]. Still, since EMT can be induced in A549 cells using TGF-β [42], they likely express less actin than fibroblasts, which explains less F-actin interactions than HDFs. Additionally, considering their smaller size compared to the HDF, mRNA localization away from the nucleus and actin-anchoring may not be as crucial as in the HDF.

Using PLA, we detected changes in the poly(A) + mRNA interactions with the cytoskeleton after F-actin depolymerization and arsenite-induced oxidative stress. We observed increased poly(A)+ mRNA binding to β-tubulin after F-actin depolymerization. Consistently, exposure to arsenite resulted in decreased poly(A)+ mRNA binding to F-actin and increased binding to β-tubulin within the first ten minutes. Interestingly, in both cytoD and arsenite exposure experiments, a complementary relationship between F-actin and β-tubulin was observed. When the mRNA granules separated from F-actin, their binding to microtubules increased. To date, a dynamic relationship between mRNA and cytoskeletal components has not been reported. The mechanism for the similarity of these responses is unknown. Similar to cytoD, arsenite also disrupts actin filament formation [36,37], but in a less direct and intense manner. With the arsenite exposure, mRNAs do not fall off of the F-actin, as with cytoD exposure, instead they may be moved onto microtubules predominantly and intermediate filaments to a lesser degree. This implies that the dynamic relationship between mRNA localization to F-actin and microtubules is likely a conserved response in both cytoD and arsenite exposure experiments, and not specific to F-actin depolymerization. The mechanism of this event will be explored in future publications.

In addition, these observations elicit a number of questions regarding mRNA control. The trans-acting factors controlling the mRNA localization to F-actin versus microtubules remain to be identified. The significance of mRNA binding to cytoskeleton in directing its function and fate toward translation or degradation also needs to be elucidated. Are the mRNAs switching from F-actin to microtubules so that they can be degraded? Or are they simply binding to microtubules randomly, because they are no longer bound to F-actin? Future studies, using the method discussed in this paper in conjunction with other methods, such as crosslinking and immunoprecipitation, should allow us to answer these questions.

Here, we present a powerful tool for imaging and quantifying mRNA-cytoskeleton interactions. It has shown to provide similar accuracy as EM but is easier to perform and is less labor-intensive. Unlike other methods, this technique can quantify the spatiotemporal asymmetry within an individual and population of cells as well as cell-to-cell variations, which are valuable in numerous studies, such as viral infection [43-45], stem cell differentiation [46-48], and cancer pathophysiology [49-51]. This method, in future studies, will assist in improving our understanding of the effects of intracellular and extracellular signaling events on mRNA trafficking, localization, and translation via alterations in the interactions between the mRNA, RBPs, motor proteins, and cytoskeleton. Multiplexing this assay to detect multiple RNA-cytoskeleton and RNA-RBP interactions, simultaneously, will provide a more complete understanding of how these interactions are altered during infections, oxidative stress, or during the application of external forces. By combining this method with other assays, such as immunoprecipitation, that can identify RBPs that regulate mRNA-cytoskeleton interactions, we can elucidate the underlying mechanism that direct mRNA localization.

## Supporting Information

Figure S1 **All and single image planes of interactions between poly(A)+ mRNA and phalloidin in human dermal fibroblasts (HDF).** Phalloidin, poly(A)+ mRNA, and proximity ligation assay (PLA) product between poly(A)+ mRNA and α-tubulin were imaged with a widefield microscope. Merged images of phalloidin (white), poly(A)+ mRNA (red), PLA (green), and nuclei (blue) are shown. All (All Planes) and single image planes (Single Plane) are represented. Scale bar, 10µm.
(TIF)

Figure S2 **Interactions between poly(A)+ mRNA and α-tubulin in A549 cells fixed with methanol, 1% paraformaldehyde (PFA) in PBS, and 1% PFA in BRB80.** α-tubulin immunofluorescence (IF), poly(A)+ mRNA, and proximity ligation assay (PLA) product between poly(A)+ mRNA and α-tubulin were imaged with a laser-scanning confocal microscope. Merged images of α-tubulin (white), poly(A)+ mRNA (red), PLA (green), and nuclei (blue) are shown. Single image plane is represented. Scale bar, 10µm.
(TIF)

Figure S3 **Interactions between poly(A)+ mRNA and vimentin in A549 cells fixed with methanol, 1% PFA in PBS, and 1% PFA in BRB80.** (**A**) Vimentin IF, poly(A)+ mRNA, and PLA product between poly(A)+ mRNA and vimentin were imaged with a laser-scanning confocal microscope. Merged images of Vimentin (white), poly(A)+ mRNA (red), PLA (green), and nuclei (blue) are shown. Single image plane is represented. Scale bar, 10 µm. (B) The mean FMTRIP volume was similar (Kruskal-Wallis One Way ANOVA on Ranks, p=0.5) in all fixatives, methanol (n=20), 1% PFA in PBS (n=20), and 1% PFA in BRB80 (n=21). (C) The mean percentage of FMTRIP colocalized with PLA (PLA-FMTRIP) in cells fixed with methanol (n=20) was greater (Kruskal-Wallis One Way ANOVA on Ranks with Dunn’s method, ***, p<0.001) than those fixed with 1% PFA in PBS (n=20) or 1% PFA in BRB80 (n=21). (D) The mean PLA frequency was greater (Kruskal-Wallis One Way ANOVA on Ranks with Dunn’s method, *, p<0.02) in methanol fixation (n=20) than in 1% PFA in PBS (n=20) or 1% PFA in BRB80 (n=21).
(TIF)

Figure S4 **Interactions between poly(A)+ mRNA and cytoskeletal elements in A549 cells post-depolymerization of microtubules using nocodazole, intermediate filaments using acrylamide, and actin using cytochalasin D.** β-tubulin, vimentin, and phalloidin immunofluorescence (IF), poly(A)+ mRNA, and PLA between poly(A)+ mRNA and the cytoskeletal elements in HDF were imaged with a laser-scanning confocal microscope. Merged images of the cytoskeleton (white), poly(A)+ mRNA (red), PLA (green) and nuclei (blue) are shown. Single image plane is represented. Scale bar, 10 µm.
(TIF)

Figure S5 **Interactions between poly(A)+ mRNA and cytoskeletal elements in A549 cells.** (**A**) β-tubulin, vimentin and phalloidin IF, poly(A)+ mRNA, and PLA between poly(A)+ mRNA and the cytoskeletal elements in A549 cells were imaged with a laser-scanning confocal microscope. Merged images of the cytoskeleton (white), poly(A)+ mRNA (red), PLA (green) and nuclei (blue) are shown. Single image plane is represented. Inset, images of boxed regions. Scale bar, 10 µm (2 µm in insets). (**B**) The mean FMTRIP volume was similar (One Way ANOVA with normal distribution, p=0.7) in cells, where the interactions between poly(A)+ mRNA and β-tubulin (n=26, mean=146µm^3^, s.d.=42µm^3^), vimentin (n=28, mean=160µm^3^, s.d.=43µm^3^), or phalloidin (n=24, mean=177µm^3^, s.d.=51µm^3^) were quantified. (**C**) The mean percentage of FMTRIP colocalized with PLA (PLA-FMTRIP) was different (Table S3) between the interactions of poly(A)+ mRNA with β-tubulin (n=26, mean=2.1%, s.d.=1.4%), vimentin (n=28, mean=10.5%, s.d.=6.1%), or phalloidin (n=24, mean=22.9%, s.d.=18.6%). (**D**) The mean PLA frequency was significantly different (Table S4) between the interactions of poly(A)+ mRNA with β-tubulin (n=26, mean=0.063µm^-3^, s.d.=0.023µm^-3^), vimentin (n=28, mean=0.15µm^-3^, s.d.=0.07µm^-3^), or phalloidin (n=24, mean=0.13µm^-3^, s.d.=0.09µm^-3^). Error bars, s.d.
(TIF)

Figure S6 **Interactions between poly(A)+ mRNA bound to MTRIP lacking flag tag and cytoskeletal elements in A549 cells.** (**A**) β-tubulin, vimentin and phalloidin IF, poly(A)+ mRNA, and PLA between poly(A)+ mRNA and the cytoskeletal elements in HDF were imaged with a laser-scanning confocal microscope. Merged images of the cytoskeleton (white), poly(A)+ mRNA (red), PLA (green) and nuclei (blue) are shown. Single image plane is represented. Scale bar, 10 µm. (**B**) The mean MTRIP volume was similar (Kruskal-Wallis One Way ANOVA on Ranks, p=0.08) in cells, where the interactions between β-actin mRNA and β-tubulin (n=18, mean=404µm^3^, s.d.=317µm^3^), vimentin (n=19, mean=518µm^3^, s.d.=362µm^3^), or phalloidin (n=15, mean=653µm^3^, s.d.=211µm^3^) were quantified. (**C**) The mean percentage of MTRIP colocalized with PLA (PLA-MTRIP) was similarly minimal (Kruskal-Wallis One Way ANOVA on Ranks, p=0.15) in β-tubulin (n=18, mean=0.01%, s.d.=0.03%), vimentin (n=19, mean=0.04%, s.d.=0.1%), or phalloidin (n=15, mean=0.20%, s.d.=0.41%). (**D**) The mean PLA frequency was also minimal (Kruskal-Wallis One Way ANOVA on Ranks, p=0.23) in β-tubulin (n=18, mean=0.0004µm^-3^, s.d.=0.001µm^-3^), vimentin (n=19, mean=0.0008µm^-3^, s.d.=0.0001µm^-3^), or phalloidin (n=15, mean=0.001µm^-3^, s.d.=0.002µm^-3^). Error bars, s.d.
(TIF)

Figure S7 **Interactions between β-actin mRNA and cytoskeletal elements in A549 cells.** (**A**) β-tubulin, vimentin and phalloidin IF, β-actin mRNA, and PLA between β-actin mRNA and the cytoskeletal elements in A549 cells were imaged with a laser-scanning confocal microscope. Merged images of the cytoskeleton (white), β-actin mRNA (red), PLA (green) and nuclei (blue) are shown. Single image plane is represented. Inset, images of boxed regions. Scale bar, 10 µm (2 µm in insets). (**B**) The mean FMTRIP volume was similar (One Way ANOVA with normal distribution, p=0.08) in cells, where the interactions between β-actin mRNA and β-tubulin (n=31, mean=281µm^3^, s.d.=104µm^3^), vimentin (n=19, mean=336µm^3^, s.d.=114µm^3^), or phalloidin (n=20, mean=263µm^3^, s.d.=96µm^3^) were quantified. (**C**) The mean percentage of FMTRIP colocalized with PLA (PLA-FMTRIP) was significantly different (Table S7) between the interactions of β-actin mRNA with β-tubulin (n=31, mean=2.6%, s.d.=1.7%), vimentin (n=19, mean=11.8%, s.d.=5.7%), or phalloidin (n=20, mean=31.3%, s.d.=19.6%). (**D**) The mean PLA frequency for interactions between β-actin mRNA and phalloidin (n=20, mean=0.14µm^-3^, s.d.=0.08µm^-3^) was significantly greater (Table S8) than the interactions between β-actin mRNA and β-tubulin (n=31, mean=0.030µm^-3^, s.d.=0.016µm^-3^) or vimentin (n=19, mean=0.04µm^-3^, s.d.=0.03µm^-3^). Error bars, s.d.
(TIF)

Figure S8 **Interactions between β-actin mRNA bound to MTRIP lacking flag tags and cytoskeletal elements in human dermal fibroblasts (HDF).** (A) β-tubulin, vimentin, and phalloidin IF, β-actin mRNA, and PLA between β-actin mRNA and the cytoskeletal elements in HDF were imaged with a laser-scanning confocal microscope. Merged images of the cytoskeleton (white), β-actin mRNA (red), PLA (green), and nuclei (blue) are shown. Single image plane is represented. Scale bar, 10µm. (B) The mean MTRIP volume was similar (Kruskal-Wallis One Way ANOVA on Ranks, p=0.12) in cells, where the interactions between β-actin mRNA and β-tubulin (n=25, mean=750µm^3^, s.d.=460µm^3^), vimentin (n=22, mean=980µm^3^, s.d.=470µm^3^), or phalloidin (n=17, mean=710µm^3^, s.d.=340µm^3^) were quantified. (C) The mean percentage of MTRIP colocalized with PLA (PLA-MTRIP) was similarly minimal (Kruskal-Wallis One Way ANOVA on Ranks, p>0.05) in β-tubulin (n=25, mean=0.2%, s.d.=0.9%), vimentin (n=22, mean=0.1%, s.d.=0.4%), or phalloidin (n=17, mean=0.04%, s.d.=0.08%). (D) The mean PLA frequency was also minimal (Kruskal-Wallis One Way ANOVA on Ranks, p>0.05) in β-tubulin (n=25, mean=0.0001µm^-3^, 0.0003µm^-3^), vimentin (n=22, mean=0.0002µm^-3^, 0.0004µm^-3^), or phalloidin (n=17, mean=0.001µm^-3^, 0.002µm^-3^). Error bars, s.d.
(TIF)

Figure S9 **Interactions between β-actin mRNA bound to MTRIP lacking flag tags and cytoskeletal elements in A549 cells.** (A) β-tubulin, vimentin, and phalloidin IF, β-actin mRNA, and PLA between β-actin mRNA and the cytoskeletal elements in A549 cells were imaged with a laser-scanning confocal microscope. Merged images of the cytoskeleton (white), β-actin mRNA (red), PLA (green), and nuclei (blue) are shown. Single image plane is represented. Scale bar, 10µm. (B) The mean MTRIP volume was similar (Kruskal-Wallis One Way ANOVA on Ranks, p=0.08) in cells, where the interactions between β-actin mRNA and β-tubulin (n=15, mean=289µm^3^, s.d.=68µm^3^), vimentin (n=11, mean=299µm^3^, s.d.=71µm^3^), or phalloidin (n=13, mean=243µm^3^, s.d.=53µm^3^) were quantified. (C) The mean percentage of MTRIP colocalized with PLA (PLA-MTRIP) was similarly minimal (Kruskal-Wallis One Way ANOVA on Ranks, p>0.05) in β-tubulin (n=15, mean=0.0%, s.d.=0.0%), vimentin (n=11, mean=0.07%, s.d.=0.14%), or phalloidin (n=13, mean=0.003%, s.d.=0.009%). (D) The mean PLA frequency was also minimal (Kruskal-Wallis One Way ANOVA on Ranks, p>0.05) in β-tubulin (n=15, mean=0.00µm^-3^, s.d.=0.00µm^-3^), vimentin (n=11, mean=0.001µm^-3^, s.d.=0.002µm^-3^), or phalloidin (n=13, mean=0.0003µm^-3^, s.d.=0.0011µm^-3^). Error bars, s.d.
(TIF)

Figure S10 **Interactions between β-actin mRNA and nuclear proteins in A549 cells.** Nucleolin (C23) and Histone H1 IF, β-actin mRNA, and PLA between β-actin mRNA and the nuclear proteins in A549 cells were imaged with a laser-scanning confocal microscope. Merged images of the nuclear protein (white), β-actin mRNA (red), PLA (green), and nuclei (blue) are shown. All image planes are represented. Scale bar, 10µm.
(TIF)

Figure S11 **Interactions between poly(A)+ mRNA and β-tubulin at 5, 10, 20, and 40 min of arsenite exposure.** β-tubulin IF, poly(A)+ mRNA, and PLA between poly(A)+ mRNA and β-tubulin were imaged with a laser-scanning confocal microscope. Merged images of β-tubulin (white), poly(A)+ mRNA (red), PLA (green), and nuclei (blue) are shown. All image planes are represented. Scale bar, 10µm.
(TIF)

Figure S12 **Interactions between poly(A)+ mRNA and vimentin at 5, 10, 20, and 40 min of arsenite exposure.** Vimentin IF, poly(A)+ mRNA, and PLA between poly(A)+ mRNA and vimentin were imaged with a laser-scanning confocal microscope. Merged images of vimentin (white), poly(A)+ mRNA (red), PLA (green), and nuclei (blue) are shown. All image planes are represented. Scale bar, 10µm.
(TIF)

Figure S13 **Interactions between poly(A)+ mRNA and phalloidin at 5, 10, 20, and 40 min of arsenite exposure.** Phalloidin IF, poly(A)+ mRNA, and PLA between poly(A)+ mRNA and Phalloidin were imaged with a laser-scanning confocal microscope. Merged images of phalloidin (white), poly(A)+ mRNA (red), PLA (green), and nuclei (blue) are shown. All image planes are represented. Scale bar, 10µm.
(TIF)

Table S1 **Comparisons of the mean percentage of FMTRIP colocalized with PLA (PLA-FMTRIP) in human dermal fibroblasts (HDF) for the interactions between poly(A)+ mRNA and β-tubulin (n=25, mean=2.3%, SD=1.5%), vimentin (n=33, mean=11.5%, SD=9.7%), or phalloidin (n=23, mean=53.7%, SD=19.9%) using Kruskal-Wallis one-way ANOVA on ranks with Dunn’s method for multiple comparison (p < 0.001).**
(TIF)

Table S2 **Comparisons of the mean PLA frequency in HDF for the interactions between poly(A)+ mRNA and β-tubulin (n=25, mean=0.010µm^-3^, s.d.=0.007µm^-3^), vimentin (n=33, mean=0.019µm^-3^, s.d.=0.016µm^-3^), or phalloidin (n=23, mean=0.069µm^-3^, s.d.=0.039µm^-3^) using Kruskal-Wallis one-way ANOVA on ranks with Dunn’s method for multiple comparison (p < 0.001).**
(TIF)

Table S3 **Comparisons of the mean percentage of FMTRIP colocalized with PLA (PLA-FMTRIP) in A549 cells for the interactions between poly(A)+ mRNA and β-tubulin (n=26, mean=2.1%, s.d.=1.4%), vimentin (n=28, mean=10.5%, s.d.=6.1%), or phalloidin (n=24, mean=22.9%, s.d.=18.6%) using Kruskal-Wallis one-way ANOVA on ranks with Dunn’s method for multiple comparison (p < 0.001).**
(TIF)

Table S4 **Comparisons of the mean PLA frequency in A549 cells for the interactions between poly(A)+ mRNA and β-tubulin (n=26, mean=0.063µm^-3^, s.d.=0.023µm^-3^), vimentin (n=28, mean=0.15µm^-3^, s.d.=0.07µm^-3^), or phalloidin (n=24, mean=0.13µm^-3^, s.d.=0.09µm^-3^) using Kruskal-Wallis one-way ANOVA on ranks with Dunn’s method for multiple comparison (p < 0.001).**
(TIF)

Table S5 **Comparisons of the mean percentage of FMTRIP colocalized with PLA (PLA-FMTRIP) in HDF for the interactions between β-actin mRNA and β-tubulin (n=40, mean=3.9%, s.d.=3.2%), vimentin (n=36, mean=12.7%, s.d.=7.9%), or phalloidin (n=30, mean=71.5%, s.d.=20.1%) using Kruskal-Wallis one-way ANOVA on ranks with Dunn’s method for multiple comparison (p < 0.001).**
(TIF)

Table S6 **Comparisons of the mean PLA frequency in HDF for the interactions between β-actin mRNA and β-tubulin (n=40, mean=0.012µm^-3^, s.d.=0.011µm^-3^), vimentin (n=36, mean=0.027µm^-3^, s.d.=0.023µm^-3^), or phalloidin (n=30, mean=0.33µm^-3^, s.d.=0.17µm^-3^) using Kruskal -Wallis one-way ANOVA on ranks with Dunn’s method for multiple comparison (p < 0.001).**
(TIF)

Table S7 **Comparisons of the mean percentage of FMTRIP colocalized with PLA (PLA-FMTRIP) in A549 cells for the interactions between β-actin mRNA and β-tubulin (n=31, mean=2.6%, s.d.=1.7%), vimentin (n=19, mean=11.8%, s.d.=5.7%), or phalloidin (n=20, mean=31.3%, s.d.=19.6%) using Kruskal-Wallis one-way ANOVA on ranks with Dunn’s method for multiple comparison (p < 0.001).**
(TIF)

Table S8 **Comparisons of the mean PLA frequency in A549 cells for the interactions between β-actin mRNA and β-tubulin (n=31, mean=0.030µm^-3^, s.d.=0.016µm^-3^), vimentin (n=19, mean=0.04µm^-3^, s.d.=0.03µm^-3^), or phalloidin (n=20, mean=0.14µm^-3^, s.d.=0.08µm^-3^) using Kruskal-Wallis one-way ANOVA on ranks with Dunn’s method for multiple comparison (p < 0.001).**
(TIF)

Table S9 **Comparisons of the mean percentage of FMTRIP colocalized with PLA (PLA-FMTRIP) for poly(**A**) + mRNA interactions with β-tubulin in HDF cells exposed to arsenite for 0 (n=28, mean=2.5%, s.d.=1.6%), 5** (n=31, mean=12.8%,s.d.=14.6%), 10 (n=26, mean=11.3%, s.d.=9.1%), 20 (n=22, mean=2.6%, s.d.=3.8%), and 40 min (n=20, mean=2.2%, s.d.=3.0%) using Kruskal-Wallis one-way ANOVA on ranks with Dunn’s method for multiple comparison (p<0.001).
(TIF)

Table S10 **Comparisons of the mean percentage of FMTRIP colocalized with PLA (PLA-FMTRIP) for poly(**A**) + mRNA interactions with vimentin in HDF cells exposed to arsenite for 0 (n=42, mean=11.1%, s.d.=8.8%), 5** (n=23, mean=13.9%,s.d.=6.5%), 10 (n=19, mean=18.7%, s.d.=7.5%), 20 (n=13, mean=13.3%, s.d.=7.1%), and 40 min (n=17, mean=5.9%, s.d.=4.4%) using Kruskal-Wallis one-way ANOVA on ranks with Dunn’s method for multiple comparison (p<0.001).
(TIF)

Table S11 **Comparisons of the mean percentage of FMTRIP colocalized with PLA (PLA-FMTRIP) for poly(**A**) + mRNA interactions with F-actin in HDF cells exposed to arsenite for 0 (n=38, mean=49.3%, s.d.=19.4%), 5** (n=25, mean=6.0%,s.d.=5.2%), 10 (n=21, mean=9.6%, s.d.=6.4%), 20 (n=19, mean=15.4%, s.d.=9.4%), and 40 min (n=16, mean=6.8%, s.d.=4.4%) using Kruskal-Wallis one-way ANOVA on ranks with Dunn’s method for multiple comparison (p<0.001).
(TIF)

Table S12 **Poly(A) + and β-actin mRNA targeting probe sequences and modifications.**
(TIF)

## References

[1] Martin KC, Ephrussi A (2009) mRNA localization: gene expression in the spatial dimension. Cell 136: 719-730. doi:10.1016/j.cell.2009.01.044. PubMed: 19239891. 19239891 10.1016/j.cell.2009.01.044 PMC2819924

[2] Xing L, Bassell GJ (2013) mRNA localization: an orchestration of assembly, traffic and synthesis. Traffic 14: 2-14. PubMed: 22913533. 22913533 10.1111/tra.12004 PMC3548013

[3] Pillai RS, Bhattacharyya SN, Filipowicz W (2007) Repression of protein synthesis by miRNAs: how many mechanisms? Trends Cell Biol 17: 118-126. doi:10.1016/j.tcb.2006.12.007. PubMed: 17197185. 17197185 10.1016/j.tcb.2006.12.007

[4] Hill MA, Gunning P (1993) Beta and gamma actin mRNAs are differentially located within myoblasts. J Cell Biol 122: 825-832. doi:10.1083/jcb.122.4.825. PubMed: 8349732. 8349732 10.1083/jcb.122.4.825 PMC2119594

[5] Rodriguez AJ, Shenoy SM, Singer RH, Condeelis J (2006) Visualization of mRNA translation in living cells. J Cell Biol 175: 67-76. doi:10.1083/jcb.200512137. PubMed: 17030983. 17030983 10.1083/jcb.200512137 PMC2064499

[6] Condeelis J, Singer RH (2005) How and why does β-actin mRNA target? Biol Cell 97: 97-110. doi:10.1042/BC20040063. PubMed: 15601261. 15601261 10.1042/BC20040063

[7] Sundell CL, Singer RH (1991) Requirement of microfilaments in sorting of acting messenger RNA. Science 253: 1275-1277. doi:10.1126/science.1891715. PubMed: 1891715. 1891715 10.1126/science.1891715

[8] Hill MA, Schedlich L, Gunning P (1994) Serum-induced signal transduction determines the peripheral location of β-actin mRNA within the cell. J Cell Biol 126: 1221-1229. doi:10.1083/jcb.126.5.1221. PubMed: 8063859. 8063859 10.1083/jcb.126.5.1221 PMC2120154

[9] Glotzer JB, Saffrich R, Glotzer M, Ephrussi A (1997) Cytoplasmic flows localize injected oskar RNA in Drosophila oocytes. Curr Biol 7: 326-337. doi:10.1016/S0960-9822(06)00156-4. PubMed: 9115398. 9115398 10.1016/s0960-9822(06)00156-4

[10] Hesketh JE, Campbell GP, Whitelaw PF (1991) c-myc mRNA in cytoskeletal-bound polysomes in fibroblasts. Biochem J 274: 607-609. PubMed: 2006923. 2006923 10.1042/bj2740607 PMC1150182

[11] Bassell GJ, Powers CM, Taneja KL, Singer RH (1994) Single mRNAs visualized by ultrastructural in situ hybridization are principally localized at actin filament intersections in fibroblasts. J Cell Biol 126: 863-876. doi:10.1083/jcb.126.4.863. PubMed: 7914201. 7914201 10.1083/jcb.126.4.863 PMC2120111

[12] Takizawa PA, Sil A, Swedlow JR, Herskowitz I, Vale RD (1997) Actin-dependent localization of an RNA encoding a cell-fate determinant in yeast. Nature 389: 90-93. doi:10.1038/38015. PubMed: 9288973. 9288973 10.1038/38015

[13] Beach DL, Salmon ED, Bloom K (1999) Localization and anchoring of mRNA in budding yeast. Curr Biol 9: 569-578. doi:10.1016/S0960-9822(99)80260-7. PubMed: 10359695. 10359695 10.1016/s0960-9822(99)80260-7

[14] Theurkauf WE, Smiley S, Wong ML, Alberts BM (1992) Reorganization of the cytoskeleton during Drosophil. Development 115: 923-936. PubMed: 1451668. 1451668 10.1242/dev.115.4.923

[15] Shulman JM, Benton R, St Johnston D (2000) The Drosophila homolog of C-elegans PAR-1 organizes the oocyte cytoskeleton and directs oskar mRNA localization to the posterior pole. Cell 101: 377-388. doi:10.1016/S0092-8674(00)80848-X. PubMed: 10830165. 10830165 10.1016/s0092-8674(00)80848-x

[16] Bassell GJ, Zhang H, Byrd AL, Femino AM, Singer RH et al. (1998) Sorting of β-actin mRNA and protein to neurites and growth cones in culture. J Neurosci 18: 251-265. PubMed: 9412505. 9412505 10.1523/JNEUROSCI.18-01-00251.1998 PMC6793411

[17] Wu KY, Hengst U, Cox LJ, Macosko EZ, Jeromin A et al. (2005) Local translation of RhoA regulates growth cone collapse. Nature 436: 1020-1024. doi:10.1038/nature03885. PubMed: 16107849. 16107849 10.1038/nature03885 PMC1317112

[18] Lifland AW, Zurla C, Yu J, Santangelo PJ (2011) Dynamics of native β-actin mRNA transport in the cytoplasm. Traffic 12: 1000-1011. doi:10.1111/j.1600-0854.2011.01209.x. PubMed: 21518164. 21518164 10.1111/j.1600-0854.2011.01209.x PMC3134163

[19] Hesketh JE, Pryme IF (1991) Interaction between mRNA, ribosomes and the cytoskeleton. Biochem J 277: 1-10. PubMed: 1854327. 1854327 10.1042/bj2770001 PMC1151183

[20] Jansen RP (2001) mRNA localization: message on the move. Nat Rev Mol Cell Biol 2: 247-256. doi:10.1038/35067016. PubMed: 11283722. 11283722 10.1038/35067016

[21] Jambhekar A, DeRisi JL (2007) Cis-acting determinants of asymmetric, cytoplasmic RNA transport. RNA 13: 625-642. doi:10.1261/rna.262607. PubMed: 17449729. 17449729 10.1261/rna.262607 PMC1852811

[22] Raju CS, Fukuda N, López-Iglesias C, Göritz C, Visa N et al. (2011) In neurons, activity-dependent association of dendritically transported mRNA transcripts with the transacting factor CBF-A is mediated by A2RE/RTS elements. Mol Biol Cell 22: 1864-1877. doi:10.1091/mbc.E10-11-0904. PubMed: 21471000. 21471000 10.1091/mbc.E10-11-0904 PMC3103402

[23] Ling SC, Fahrner PS, Greenough WT, Gelfand VI (2004) Transport of Drosophila fragile X mental retardation protein-containing ribonucleoprotein granules by kinesin-1 and cytoplasmic dynein. Proc Natl Acad Sci U S A 101: 17428-17433. doi:10.1073/pnas.0408114101. PubMed: 15583137. 15583137 10.1073/pnas.0408114101 PMC536039

[24] Jonson L, Vikesaa J, Krogh A, Nielsen LK, Hansen TvO, et al. (2007) Molecular composition of IMP1 ribonucleoprotein granules. Mol Proteomics: Cell and Publishing House 6: 798-811

[25] Swanger SA, Bassell GJ, Gross C (2011) High-resolution fluorescence in situ hybridization to detect mRNAs in neuronal compartments in vitro and in vivo. Methods Mol Biol 714: 103-123. doi:10.1007/978-1-61779-005-8_7. PubMed: 21431737. 21431737 10.1007/978-1-61779-005-8_7

[26] Bertrand E, Chartrand P, Schaefer M, Shenoy SM, Singer RH (1998) Localization of ASH1 mRNA particles in living yeast. Mol Cell 2: 437-445. doi:10.1016/S1097-2765(00)80143-4. PubMed: 9809065. 9809065 10.1016/s1097-2765(00)80143-4

[27] Zimyanin VL, Belaya K, Pecreaux J, Gilchrist MJ, Clark A et al. (2008) In vivo imaging of oskar mRNA transport reveals the mechanism of posterior localization. Cell 134: 843-853. doi:10.1016/j.cell.2008.06.053. PubMed: 18775316. 18775316 10.1016/j.cell.2008.06.053 PMC2585615

[28] Slobodin B, Gerst JE (2010) A novel mRNA affinity purification technique for the identification of interacting proteins and transcripts in ribonucleoprotein complexes. RNA 16: 2277-2290. doi:10.1261/rna.2091710. PubMed: 20876833. 20876833 10.1261/rna.2091710 PMC2957065

[29] Santangelo PJ, Lifland AW, Curt P, Sasaki Y, Bassell GJ et al. (2009) Single molecule-sensitive probes for imaging RNA in live cells. Nat Methods 6: 347-349. doi:10.1038/nmeth.1316. PubMed: 19349979. 19349979 10.1038/nmeth.1316 PMC4297622

[30] Zurla C, Lifland AW, Santangelo PJ (2011) Characterizing mRNA interactions with RNA granules during translation initiation inhibition. PLOS ONE 6: e19727. doi:10.1371/journal.pone.0019727. PubMed: 21573130. 21573130 10.1371/journal.pone.0019727 PMC3088712

[31] Jung J, Lifland AW, Zurla C, Alonas EJ, Santangelo PJ (2012) Quantifying RNA-protein interactions *in situ* using modified-MTRIPs and proximity ligation. Nucleic Acids Res 41: e12 PubMed: 22952158. 22952158 10.1093/nar/gks837 PMC3592441

[32] Söderberg O, Gullberg M, Jarvius M, Ridderstråle K, Leuchowius K-J et al. (2006) Direct observation of individual endogenous protein complexes in situ by proximity ligation. Nat Methods 3: 995-991000. doi:10.1038/nmeth947. PubMed: 17072308. 17072308 10.1038/nmeth947

[33] Carson JH, Worboys K, Ainger K, Barbarese E (1997) Translocation of myelin basic protein mRNA in oligodendrocytes requires microtubules and kinesin. Cell Motil Cytoskeleton 38: 318-328. doi:10.1002/(SICI)1097-0169(1997)38:4. PubMed: 9415374. 9415374 10.1002/(SICI)1097-0169(1997)38:4<318::AID-CM2>3.0.CO;2-#

[34] Johnsson AK, Karlsson R (2010) Microtubule-dependent localization of profilin I mRNA to actin polymerization sites in serum-stimulated cells. Eur J Cell Biol 89: 394-401. doi:10.1016/j.ejcb.2009.10.020. PubMed: 20129697. 20129697 10.1016/j.ejcb.2009.10.020

[35] Sager PR (1989) Cytoskeletal effects on acrylamide and 2,5-hexanedione: selective aggregation of vimentin filaments. Toxicol Appl Pharmacol 97: 141-155. doi:10.1016/0041-008X(89)90063-X. PubMed: 2464860. 2464860 10.1016/0041-008x(89)90063-x

[36] Chou IN (1989) Distinct cytoskeletal injuries by As, Cd, Co, Cr, and Ni compounds. Biomed Environ Sci 2: 358-365. PubMed: 2604903. 2604903

[37] Li W, Chou IN (1992) Effects of sodium arsenite on the cytoskeleton and cellular glutathione levels in cultured cells. Toxicol Appl Pharmacol 114: 132-139. doi:10.1016/0041-008X(92)90105-2. PubMed: 1585365. 1585365 10.1016/0041-008x(92)90105-2

[38] Heckman C (2008) Preparation of cultured cells for transmission electron microscope. Protocol. Exchange.

[39] Kalluri R, Neilson EG (2003) Epithelial-mesenchymal transition and its implications for fibrosis. J Clin Invest 112: 1776-1784. doi:10.1172/JCI20530. PubMed: 14679171. 14679171 10.1172/JCI20530 PMC297008

[40] Mathias RA, Simpson RJ (2009) Towards understanding epithelial-mesenchymal transition: a proteomics perspective. Biochim Biophys Acta 1794: 1325-1331. doi:10.1016/j.bbapap.2009.05.001. PubMed: 19439204. 19439204 10.1016/j.bbapap.2009.05.001

[41] Oyanagi J, Ogawa T, Sato H, Higashi S, Miyazaki K (2012) Epithelial-mesenchymal transition stimulates human cancer cells to extend microtubule-based invasive protrusions an suppresses cell growth in collagen gel. PLOS ONE 7: e53209. doi:10.1371/journal.pone.0053209. PubMed: 23300891. 23300891 10.1371/journal.pone.0053209 PMC3534040

[42] Pirozzi G, Tirino V, Camerlingo R, Franco R, La Rocca A et al. (2011) Epithelial to mesenchymal transition by TGFβ-1 induction increases stemness characteristics in primary non small cell lung cancer cell line. PLOS ONE 6: e21548. doi:10.1371/journal.pone.0021548. PubMed: 21738704. 21738704 10.1371/journal.pone.0021548 PMC3128060

[43] Heinlein M, Epel BL, Padgett HS, Beachy RN (1995) Interaction of tobamovirus movement proteins with the plant cytoskeleton. Science 270: 1983-1985. doi:10.1126/science.270.5244.1983. PubMed: 8533089. 8533089 10.1126/science.270.5244.1983

[44] Bukrinskaya A, Brichacek B, Mann A, Stevenson M (1998) Establishment of a functional human immunodeficiency virus type 1 (HIV-1) reverse transcription complex involves the cytoskeleton. J Exp Med 188: 2113-2125. doi:10.1084/jem.188.11.2113. PubMed: 9841925. 9841925 10.1084/jem.188.11.2113 PMC2212381

[45] Nemerow GR, Stewart PL (1999) Role of α_v_ integrins in adenovirus cell entry and gene delivery. Microbiol Mol Biol Rev 63: 725-734. PubMed: 10477314. 10477314 10.1128/mmbr.63.3.725-734.1999 PMC103752

[46] Suon S, Jin H, Donaldson AE, Caterson EJ, Tuan RS et al. (2004) Transient differentiation of adult human bone marrow cells into neuron-like cells in culture: development of morphological and biochemical traits is mediated by different molecular mechanisms. Stem Cells Dev 13: 625-635. doi:10.1089/scd.2004.13.625. PubMed: 15684830. 15684830 10.1089/scd.2004.13.625 PMC1976185

[47] Ratti A, Fallini C, Cova L, Fantozzi R, Calzarossa C et al. (2006) A role for the ELAV RNA-binding proteins in neural stem cells: stabilization of *Msi1* mRNA. J Cell Sci 119: 1442-1452. doi:10.1242/jcs.02852. PubMed: 16554442. 16554442 10.1242/jcs.02852

[48] Hailesellasse Sene K, Porter CJ, Palidwor G, Perez-Iratxeta C, Muro EM et al. (2007) Gene function in early mouse embryonic stem cell differentiation. BMC Genomics 8: 85. doi:10.1186/1471-2164-8-85. PubMed: 17394647. 17394647 10.1186/1471-2164-8-85 PMC1851713

[49] Zimber A, Nguyen QD, Gespach C (2004) Nuclear bodies and compartments: functional roles and cellular signalling in health and disease. Cell Signal 16: 1085-1104. doi:10.1016/j.cellsig.2004.03.020. PubMed: 15240004. 15240004 10.1016/j.cellsig.2004.03.020

[50] Greijer AE, van der Groep P, Kemming D, Shvarts A, Semenza GL et al. (2005) Up-regulation of gene expression by hypoxia is mediated predominantly by hypoxia-inducible factor 1 (HIF-1). J Pathol 206: 291-304. doi:10.1002/path.1778. PubMed: 15906272. 15906272 10.1002/path.1778

[51] Escuin D, Kline ER, Giannakakou P (2005) Both microtubule-stabilizing and microtubule-destabilizing drugs inhibit hypoxia-inducible factor-1α accumulation and activity by disrupting microtubule function. Cancer Res 65: 9021-9028. doi:10.1158/0008-5472.CAN-04-4095. PubMed: 16204076. 16204076 10.1158/0008-5472.CAN-04-4095 PMC6623969

