# Supplementary figures and images for: Characterization of mRNA-Cytoskeleton Interactions In Situ Using FMTRIP and Proximity Ligation
Source: PLoS One. 2013 Sep 11;8(9):e74598. doi: 10.1371/journal.pone.0074598 (PMC3770708; doi:10.1371/journal.pone.0074598)

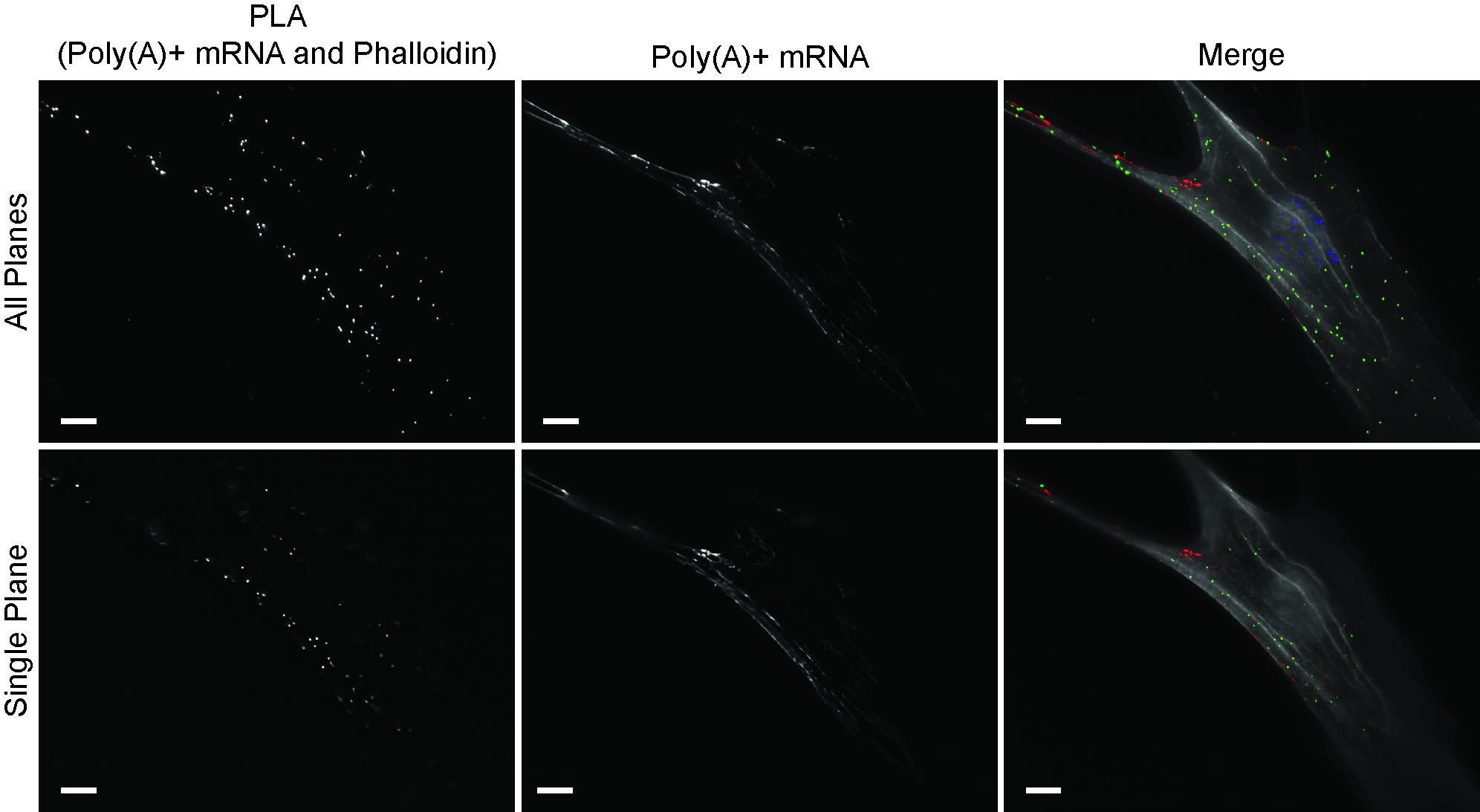

Supplement: Figure S1 — All and single image planes of interactions between poly(A)+ mRNA and phalloidin in human dermal fibroblasts (HDF). Phalloidin, poly(A)+ mRNA, and proximity ligation assay (PLA) product between poly(A)+ mRNA and α-tubulin were imaged with a widefield microscope. Merged images of phalloidin (white), poly(A)+ mRNA (red), PLA (green), and nuclei (blue) are shown. All (All Planes) and single image planes (Single Plane) are represented. Scale bar, 10µm. (TIF) [file pone.0074598.s001.tif]

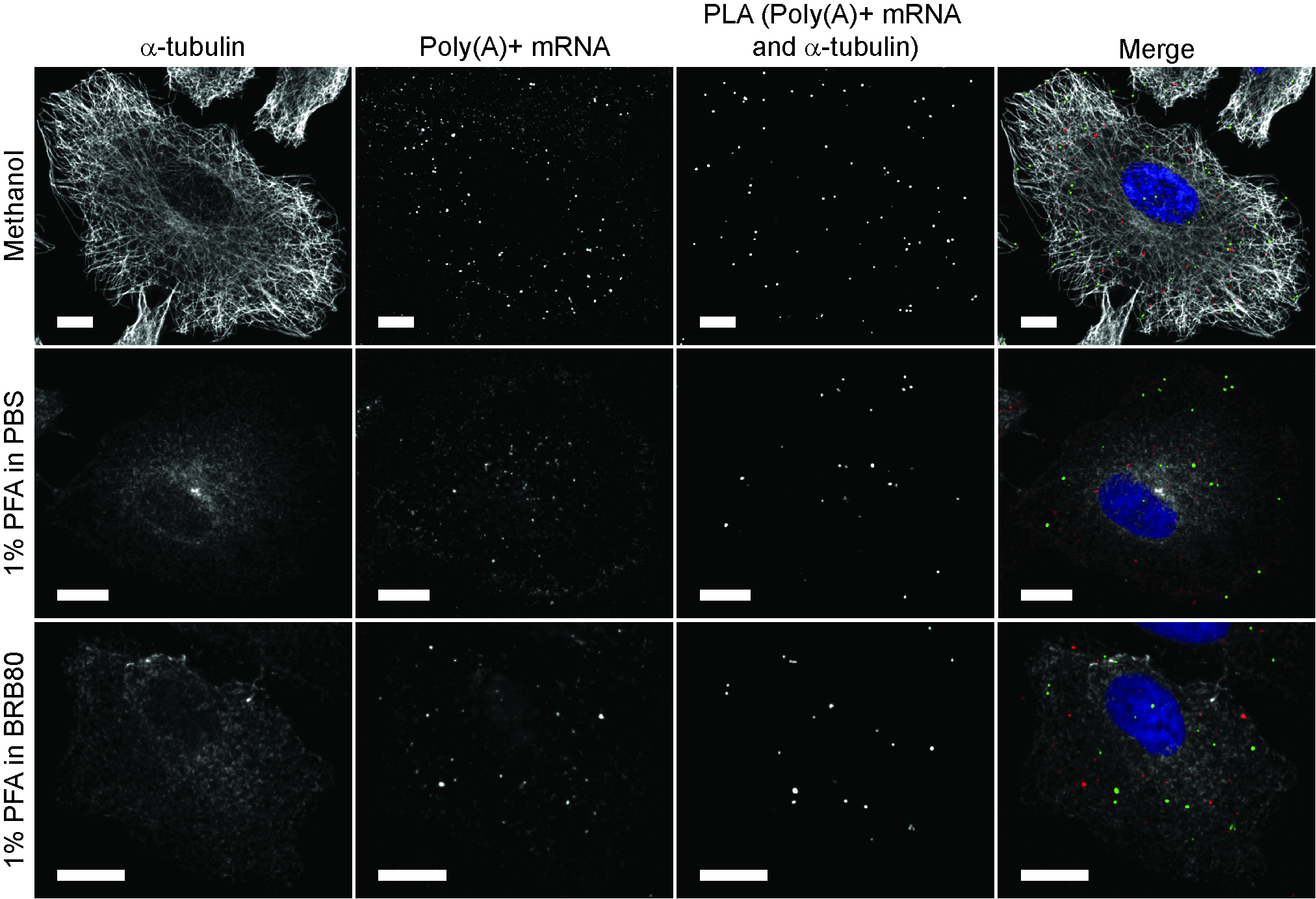

Supplement: Figure S2 — Interactions between poly(A)+ mRNA and α-tubulin in A549 cells fixed with methanol, 1% paraformaldehyde (PFA) in PBS, and 1% PFA in BRB80. α-tubulin immunofluorescence (IF), poly(A)+ mRNA, and proximity ligation assay (PLA) product between poly(A)+ mRNA and α-tubulin were imaged with a laser-scanning confocal microscope. Merged images of α-tubulin (white), poly(A)+ mRNA (red), PLA (green), and nuclei (blue) are shown. Single image plane is represented. Scale bar, 10µm. (TIF) [file pone.0074598.s002.tif]

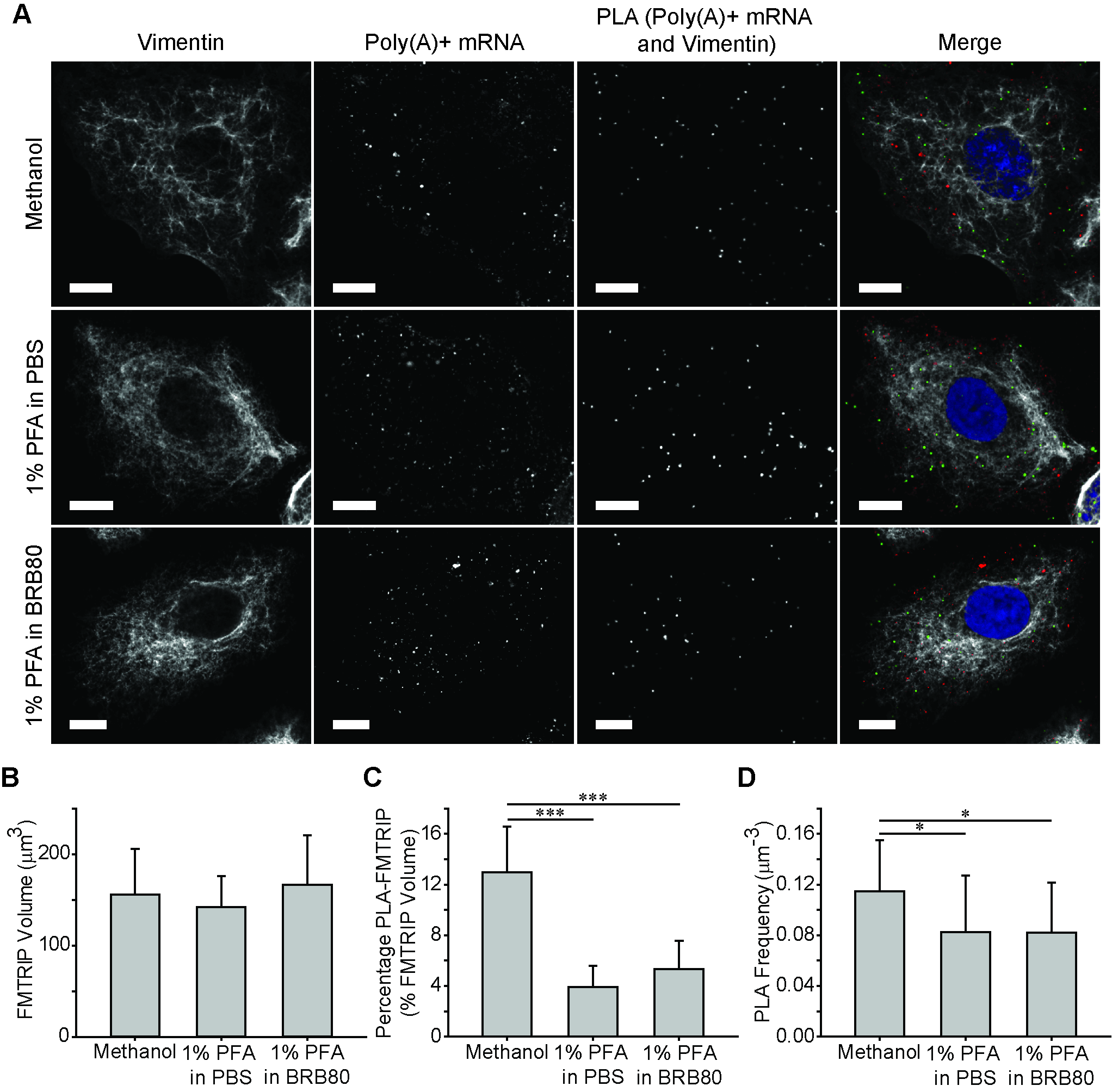

Supplement: Figure S3 — Interactions between poly(A)+ mRNA and vimentin in A549 cells fixed with methanol, 1% PFA in PBS, and 1% PFA in BRB80. (A) Vimentin IF, poly(A)+ mRNA, and PLA product between poly(A)+ mRNA and vimentin were imaged with a laser-scanning confocal microscope. Merged images of Vimentin (white), poly(A)+ mRNA (red), PLA (green), and nuclei (blue) are shown. Single image plane is represented. Scale bar, 10 µm. (B) The mean FMTRIP volume was similar (Kruskal-Wallis One Way ANOVA on Ranks, p=0.5) in all fixatives, methanol (n=20), 1% PFA in PBS (n=20), and 1% PFA in BRB80 (n=21). (C) The mean percentage of FMTRIP colocalized with PLA (PLA-FMTRIP) in cells fixed with methanol (n=20) was greater (Kruskal-Wallis One Way ANOVA on Ranks with Dunn’s method, ***, p<0.001) than those fixed with 1% PFA in PBS (n=20) or 1% PFA in BRB80 (n=21). (D) The mean PLA frequency was greater (Kruskal-Wallis One Way ANOVA on Ranks with Dunn’s method, *, p<0.02) in methanol fixation (n=20) than in 1% PFA in PBS (n=20) or 1% PFA in BRB80 (n=21). (TIF) [file pone.0074598.s003.tif]

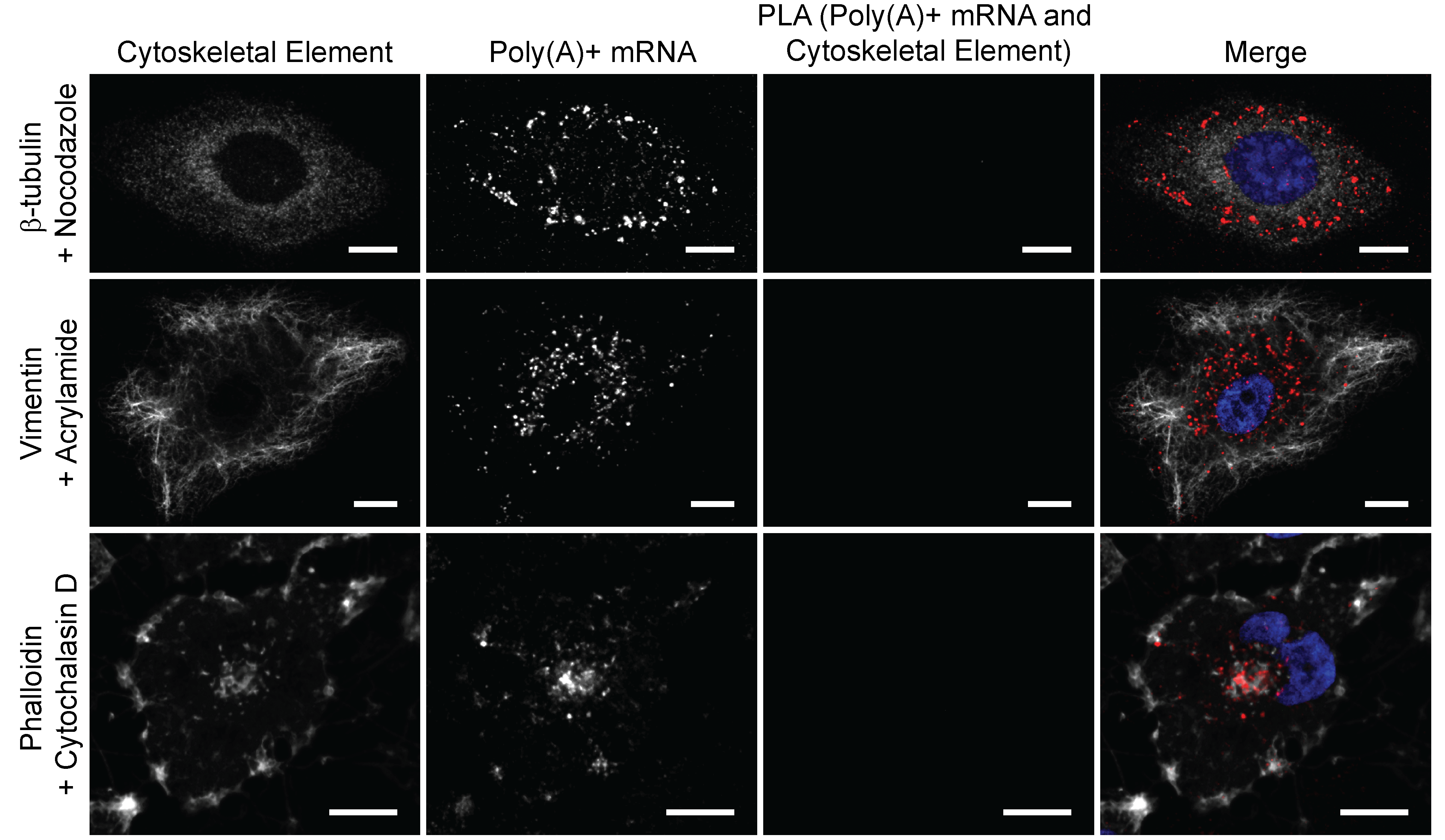

Supplement: Figure S4 — Interactions between poly(A)+ mRNA and cytoskeletal elements in A549 cells post-depolymerization of microtubules using nocodazole, intermediate filaments using acrylamide, and actin using cytochalasin D. β-tubulin, vimentin, and phalloidin immunofluorescence (IF), poly(A)+ mRNA, and PLA between poly(A)+ mRNA and the cytoskeletal elements in HDF were imaged with a laser-scanning confocal microscope. Merged images of the cytoskeleton (white), poly(A)+ mRNA (red), PLA (green) and nuclei (blue) are shown. Single image plane is represented. Scale bar, 10 µm. (TIF) [file pone.0074598.s004.tif]

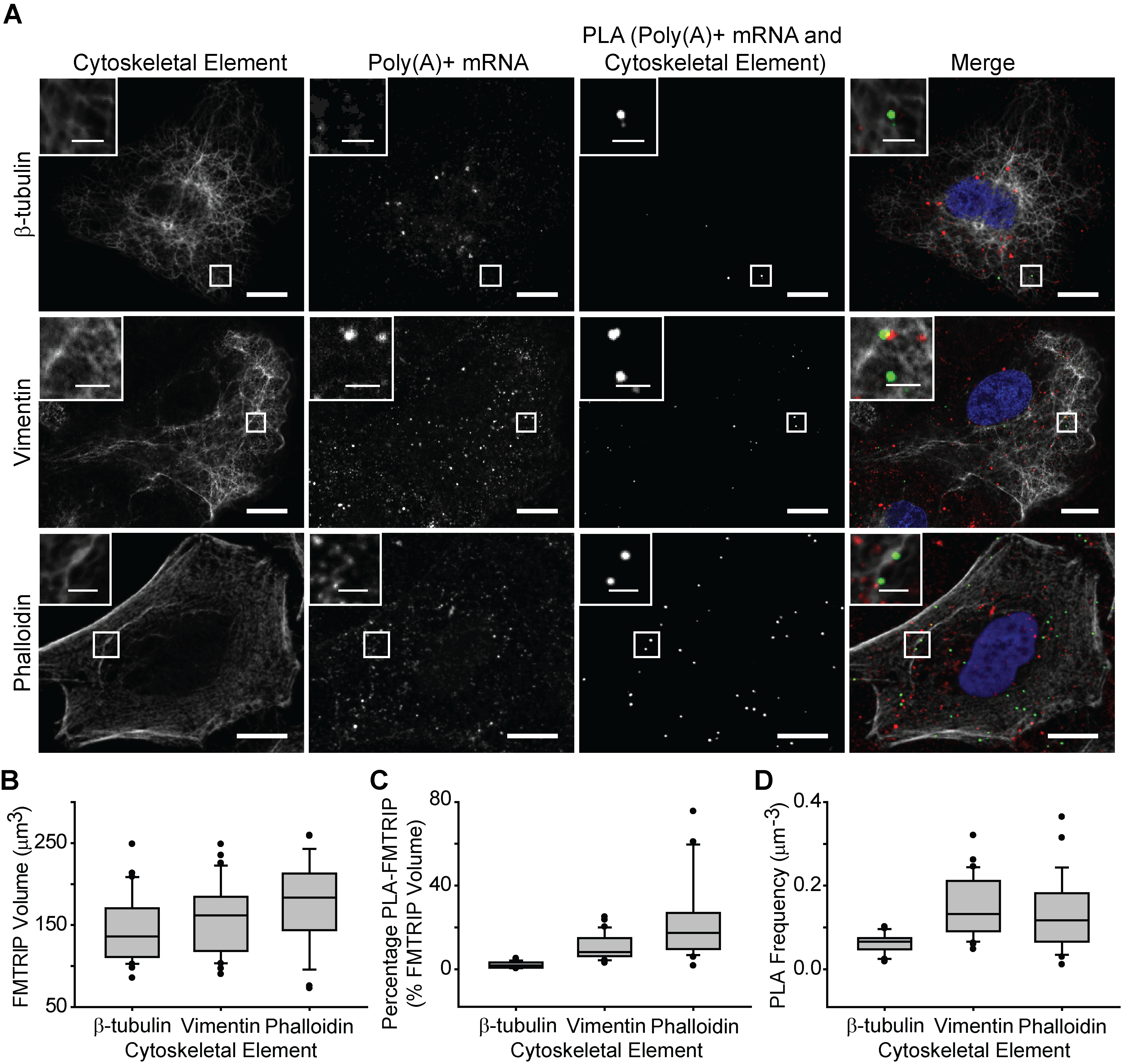

Supplement: Figure S5 — Interactions between poly(A)+ mRNA and cytoskeletal elements in A549 cells. (A) β-tubulin, vimentin and phalloidin IF, poly(A)+ mRNA, and PLA between poly(A)+ mRNA and the cytoskeletal elements in A549 cells were imaged with a laser-scanning confocal microscope. Merged images of the cytoskeleton (white), poly(A)+ mRNA (red), PLA (green) and nuclei (blue) are shown. Single image plane is represented. Inset, images of boxed regions. Scale bar, 10 µm (2 µm in insets). (B) The mean FMTRIP volume was similar (One Way ANOVA with normal distribution, p=0.7) in cells, where the interactions between poly(A)+ mRNA and β-tubulin (n=26, mean=146µm3, s.d.=42µm3), vimentin (n=28, mean=160µm3, s.d.=43µm3), or phalloidin (n=24, mean=177µm3, s.d.=51µm3) were quantified. (C) The mean percentage of FMTRIP colocalized with PLA (PLA-FMTRIP) was different (Table S3) between the interactions of poly(A)+ mRNA with β-tubulin (n=26, mean=2.1%, s.d.=1.4%), vimentin (n=28, mean=10.5%, s.d.=6.1%), or phalloidin (n=24, mean=22.9%, s.d.=18.6%). (D) The mean PLA frequency was significantly different (Table S4) between the interactions of poly(A)+ mRNA with β-tubulin (n=26, mean=0.063µm-3, s.d.=0.023µm-3), vimentin (n=28, mean=0.15µm-3, s.d.=0.07µm-3), or phalloidin (n=24, mean=0.13µm-3, s.d.=0.09µm-3). Error bars, s.d. (TIF) [file pone.0074598.s005.tif]

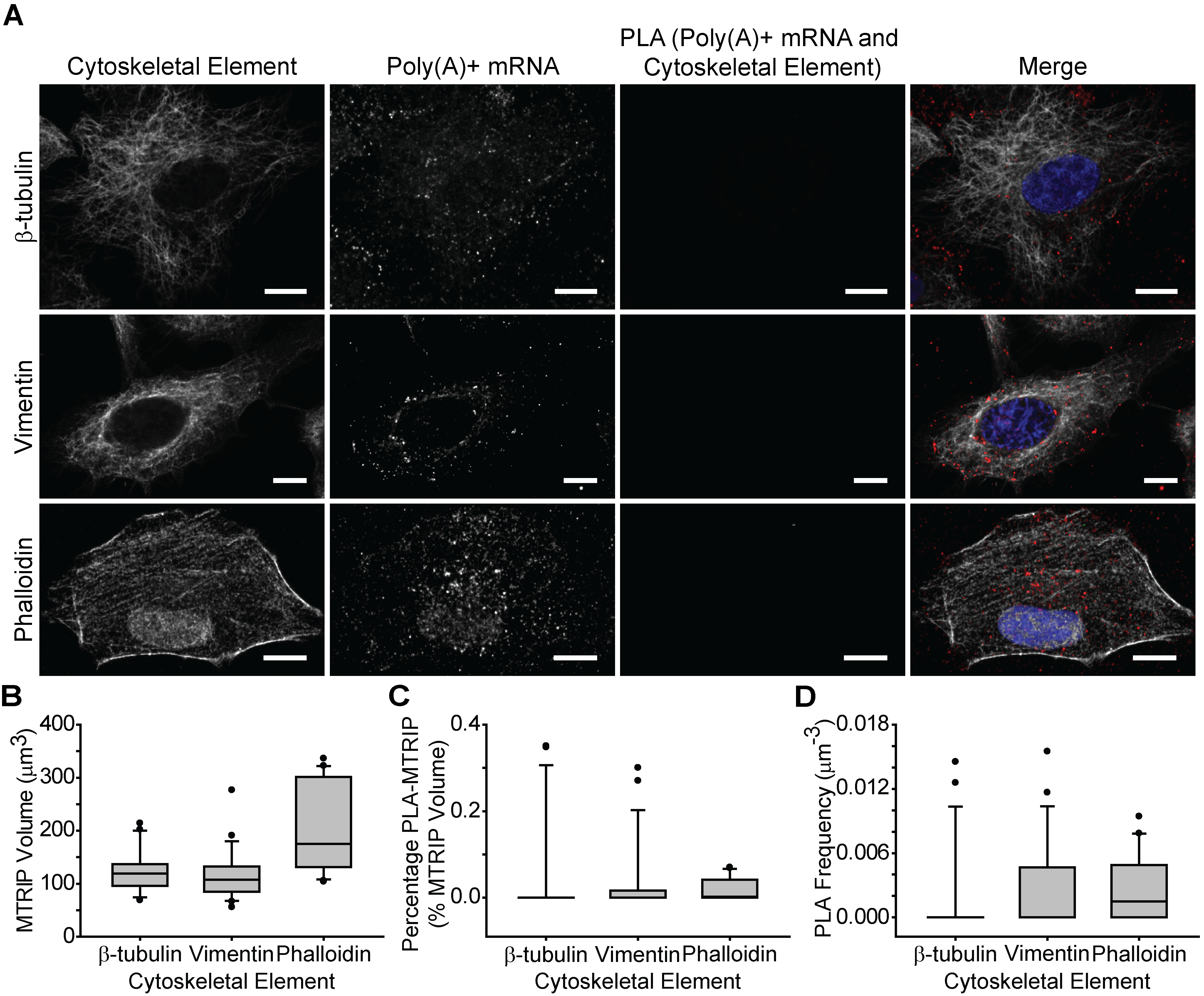

Supplement: Figure S6 — Interactions between poly(A)+ mRNA bound to MTRIP lacking flag tag and cytoskeletal elements in A549 cells. (A) β-tubulin, vimentin and phalloidin IF, poly(A)+ mRNA, and PLA between poly(A)+ mRNA and the cytoskeletal elements in HDF were imaged with a laser-scanning confocal microscope. Merged images of the cytoskeleton (white), poly(A)+ mRNA (red), PLA (green) and nuclei (blue) are shown. Single image plane is represented. Scale bar, 10 µm. (B) The mean MTRIP volume was similar (Kruskal-Wallis One Way ANOVA on Ranks, p=0.08) in cells, where the interactions between β-actin mRNA and β-tubulin (n=18, mean=404µm3, s.d.=317µm3), vimentin (n=19, mean=518µm3, s.d.=362µm3), or phalloidin (n=15, mean=653µm3, s.d.=211µm3) were quantified. (C) The mean percentage of MTRIP colocalized with PLA (PLA-MTRIP) was similarly minimal (Kruskal-Wallis One Way ANOVA on Ranks, p=0.15) in β-tubulin (n=18, mean=0.01%, s.d.=0.03%), vimentin (n=19, mean=0.04%, s.d.=0.1%), or phalloidin (n=15, mean=0.20%, s.d.=0.41%). (D) The mean PLA frequency was also minimal (Kruskal-Wallis One Way ANOVA on Ranks, p=0.23) in β-tubulin (n=18, mean=0.0004µm-3, s.d.=0.001µm-3), vimentin (n=19, mean=0.0008µm-3, s.d.=0.0001µm-3), or phalloidin (n=15, mean=0.001µm-3, s.d.=0.002µm-3). Error bars, s.d. (TIF) [file pone.0074598.s006.tif]

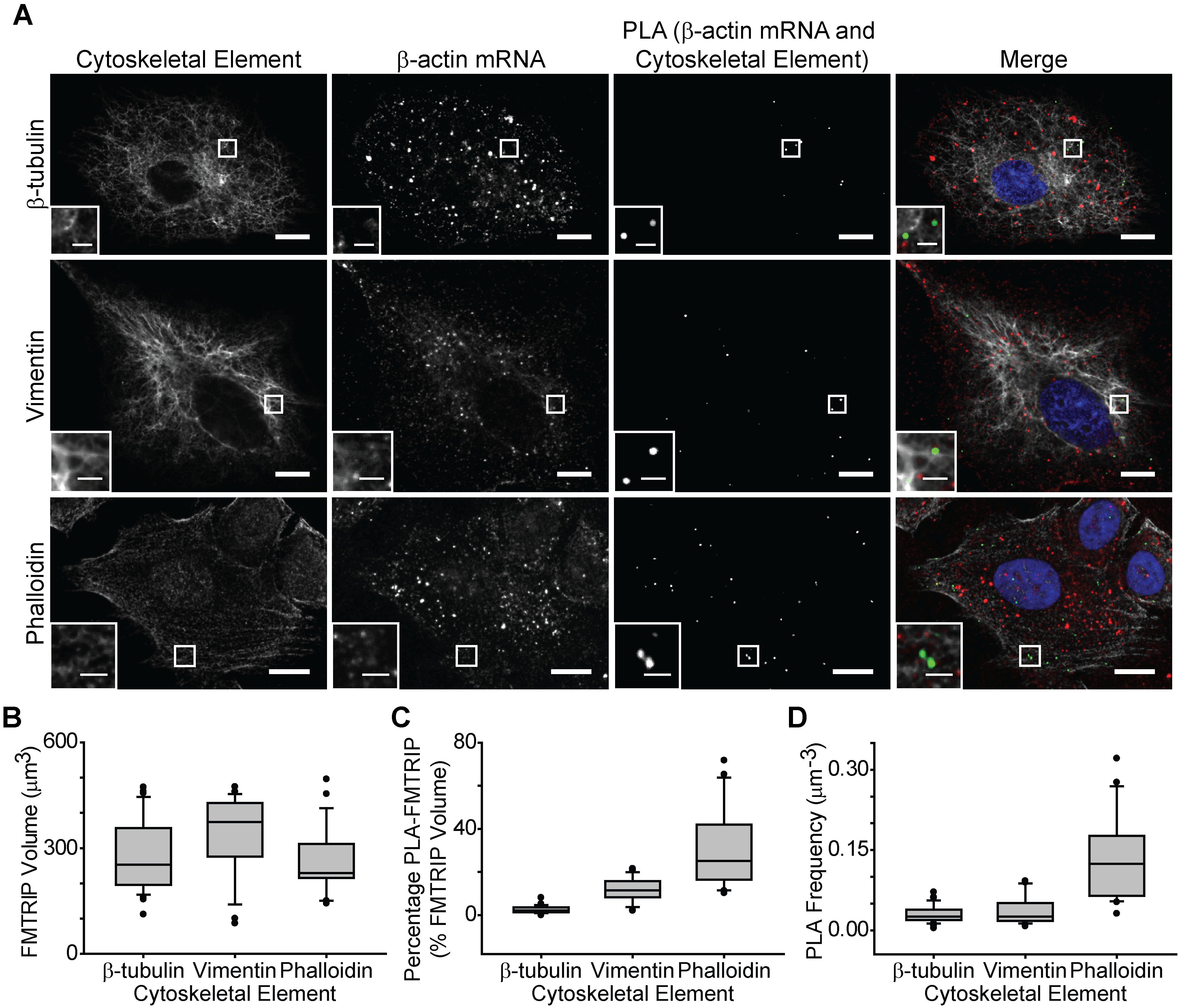

Supplement: Figure S7 — Interactions between β-actin mRNA and cytoskeletal elements in A549 cells. (A) β-tubulin, vimentin and phalloidin IF, β-actin mRNA, and PLA between β-actin mRNA and the cytoskeletal elements in A549 cells were imaged with a laser-scanning confocal microscope. Merged images of the cytoskeleton (white), β-actin mRNA (red), PLA (green) and nuclei (blue) are shown. Single image plane is represented. Inset, images of boxed regions. Scale bar, 10 µm (2 µm in insets). (B) The mean FMTRIP volume was similar (One Way ANOVA with normal distribution, p=0.08) in cells, where the interactions between β-actin mRNA and β-tubulin (n=31, mean=281µm3, s.d.=104µm3), vimentin (n=19, mean=336µm3, s.d.=114µm3), or phalloidin (n=20, mean=263µm3, s.d.=96µm3) were quantified. (C) The mean percentage of FMTRIP colocalized with PLA (PLA-FMTRIP) was significantly different (Table S7) between the interactions of β-actin mRNA with β-tubulin (n=31, mean=2.6%, s.d.=1.7%), vimentin (n=19, mean=11.8%, s.d.=5.7%), or phalloidin (n=20, mean=31.3%, s.d.=19.6%). (D) The mean PLA frequency for interactions between β-actin mRNA and phalloidin (n=20, mean=0.14µm-3, s.d.=0.08µm-3) was significantly greater (Table S8) than the interactions between β-actin mRNA and β-tubulin (n=31, mean=0.030µm-3, s.d.=0.016µm-3) or vimentin (n=19, mean=0.04µm-3, s.d.=0.03µm-3). Error bars, s.d. (TIF) [file pone.0074598.s007.tif]

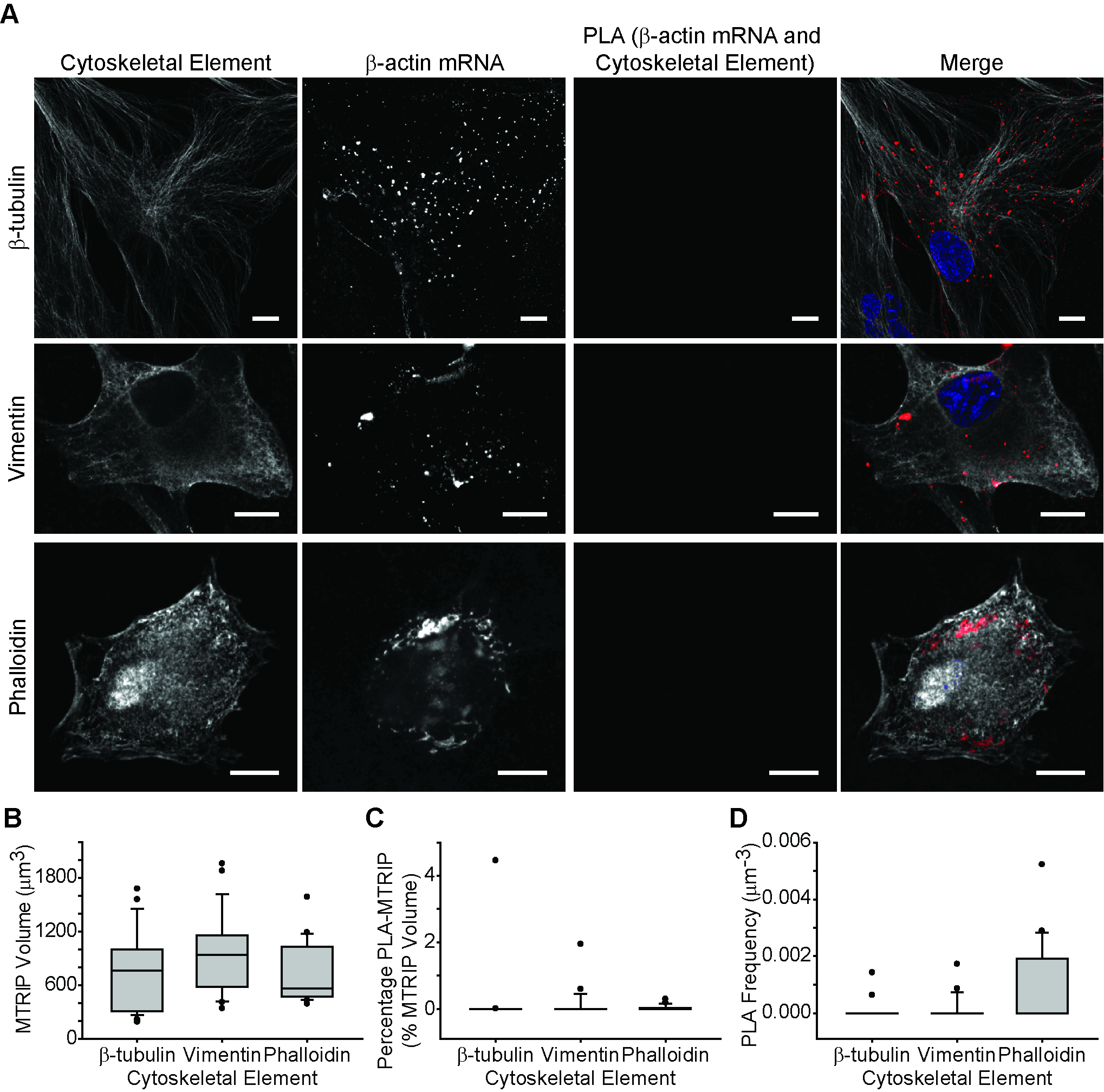

Supplement: Figure S8 — Interactions between β-actin mRNA bound to MTRIP lacking flag tags and cytoskeletal elements in human dermal fibroblasts (HDF). (A) β-tubulin, vimentin, and phalloidin IF, β-actin mRNA, and PLA between β-actin mRNA and the cytoskeletal elements in HDF were imaged with a laser-scanning confocal microscope. Merged images of the cytoskeleton (white), β-actin mRNA (red), PLA (green), and nuclei (blue) are shown. Single image plane is represented. Scale bar, 10µm. (B) The mean MTRIP volume was similar (Kruskal-Wallis One Way ANOVA on Ranks, p=0.12) in cells, where the interactions between β-actin mRNA and β-tubulin (n=25, mean=750µm3, s.d.=460µm3), vimentin (n=22, mean=980µm3, s.d.=470µm3), or phalloidin (n=17, mean=710µm3, s.d.=340µm3) were quantified. (C) The mean percentage of MTRIP colocalized with PLA (PLA-MTRIP) was similarly minimal (Kruskal-Wallis One Way ANOVA on Ranks, p>0.05) in β-tubulin (n=25, mean=0.2%, s.d.=0.9%), vimentin (n=22, mean=0.1%, s.d.=0.4%), or phalloidin (n=17, mean=0.04%, s.d.=0.08%). (D) The mean PLA frequency was also minimal (Kruskal-Wallis One Way ANOVA on Ranks, p>0.05) in β-tubulin (n=25, mean=0.0001µm-3, 0.0003µm-3), vimentin (n=22, mean=0.0002µm-3, 0.0004µm-3), or phalloidin (n=17, mean=0.001µm-3, 0.002µm-3). Error bars, s.d. (TIF) [file pone.0074598.s008.tif]

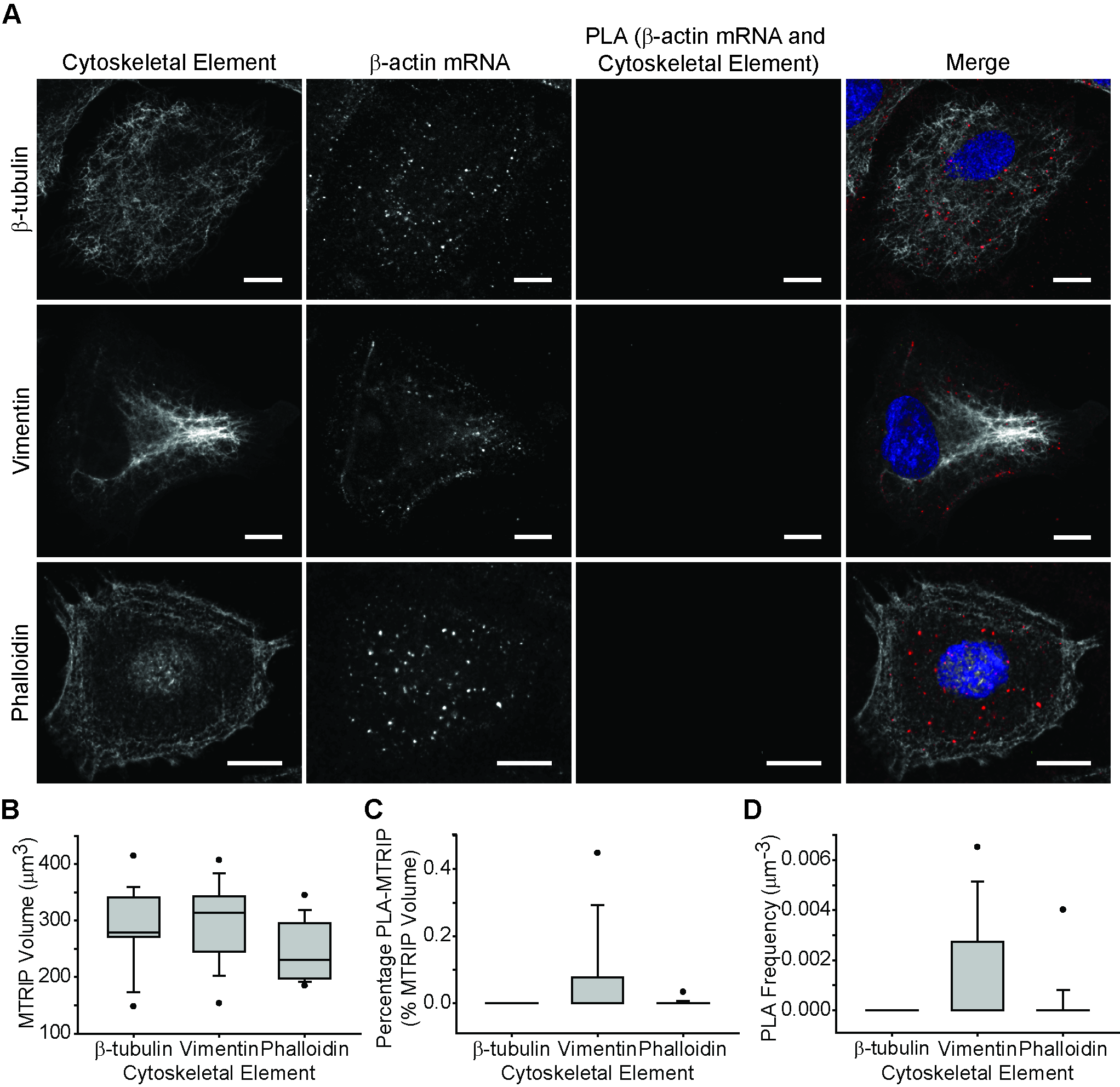

Supplement: Figure S9 — Interactions between β-actin mRNA bound to MTRIP lacking flag tags and cytoskeletal elements in A549 cells. (A) β-tubulin, vimentin, and phalloidin IF, β-actin mRNA, and PLA between β-actin mRNA and the cytoskeletal elements in A549 cells were imaged with a laser-scanning confocal microscope. Merged images of the cytoskeleton (white), β-actin mRNA (red), PLA (green), and nuclei (blue) are shown. Single image plane is represented. Scale bar, 10µm. (B) The mean MTRIP volume was similar (Kruskal-Wallis One Way ANOVA on Ranks, p=0.08) in cells, where the interactions between β-actin mRNA and β-tubulin (n=15, mean=289µm3, s.d.=68µm3), vimentin (n=11, mean=299µm3, s.d.=71µm3), or phalloidin (n=13, mean=243µm3, s.d.=53µm3) were quantified. (C) The mean percentage of MTRIP colocalized with PLA (PLA-MTRIP) was similarly minimal (Kruskal-Wallis One Way ANOVA on Ranks, p>0.05) in β-tubulin (n=15, mean=0.0%, s.d.=0.0%), vimentin (n=11, mean=0.07%, s.d.=0.14%), or phalloidin (n=13, mean=0.003%, s.d.=0.009%). (D) The mean PLA frequency was also minimal (Kruskal-Wallis One Way ANOVA on Ranks, p>0.05) in β-tubulin (n=15, mean=0.00µm-3, s.d.=0.00µm-3), vimentin (n=11, mean=0.001µm-3, s.d.=0.002µm-3), or phalloidin (n=13, mean=0.0003µm-3, s.d.=0.0011µm-3). Error bars, s.d. (TIF) [file pone.0074598.s009.tif]

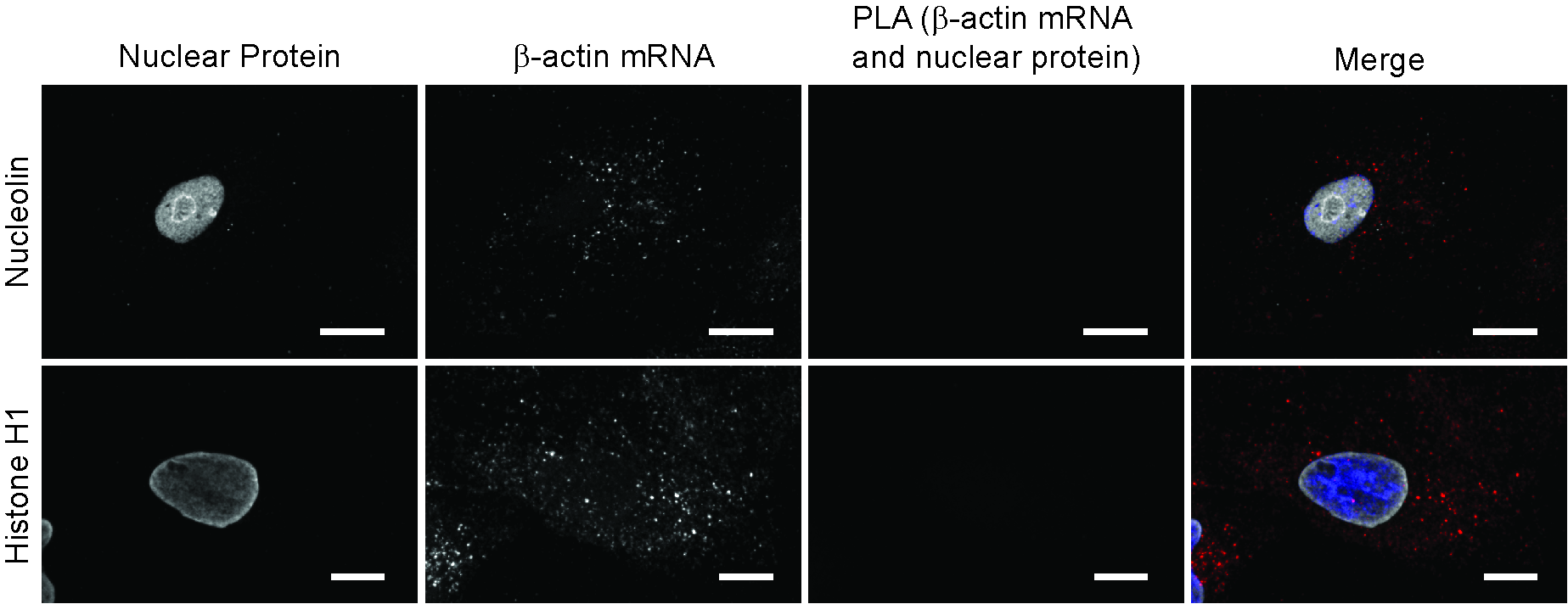

Supplement: Figure S10 — Interactions between β-actin mRNA and nuclear proteins in A549 cells. Nucleolin (C23) and Histone H1 IF, β-actin mRNA, and PLA between β-actin mRNA and the nuclear proteins in A549 cells were imaged with a laser-scanning confocal microscope. Merged images of the nuclear protein (white), β-actin mRNA (red), PLA (green), and nuclei (blue) are shown. All image planes are represented. Scale bar, 10µm. (TIF) [file pone.0074598.s010.tif]

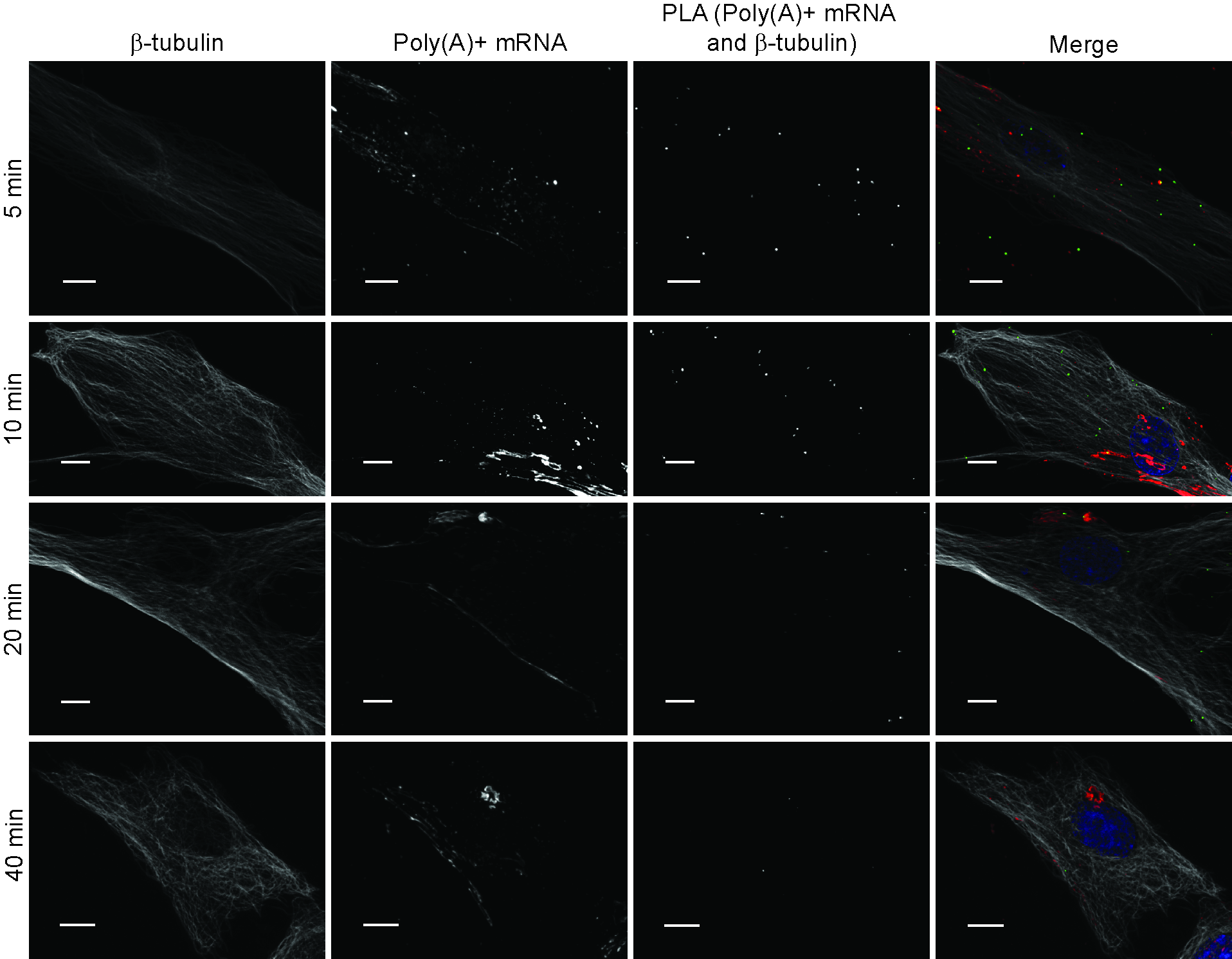

Supplement: Figure S11 — Interactions between poly(A)+ mRNA and β-tubulin at 5, 10, 20, and 40 min of arsenite exposure. β-tubulin IF, poly(A)+ mRNA, and PLA between poly(A)+ mRNA and β-tubulin were imaged with a laser-scanning confocal microscope. Merged images of β-tubulin (white), poly(A)+ mRNA (red), PLA (green), and nuclei (blue) are shown. All image planes are represented. Scale bar, 10µm. (TIF) [file pone.0074598.s011.tif]

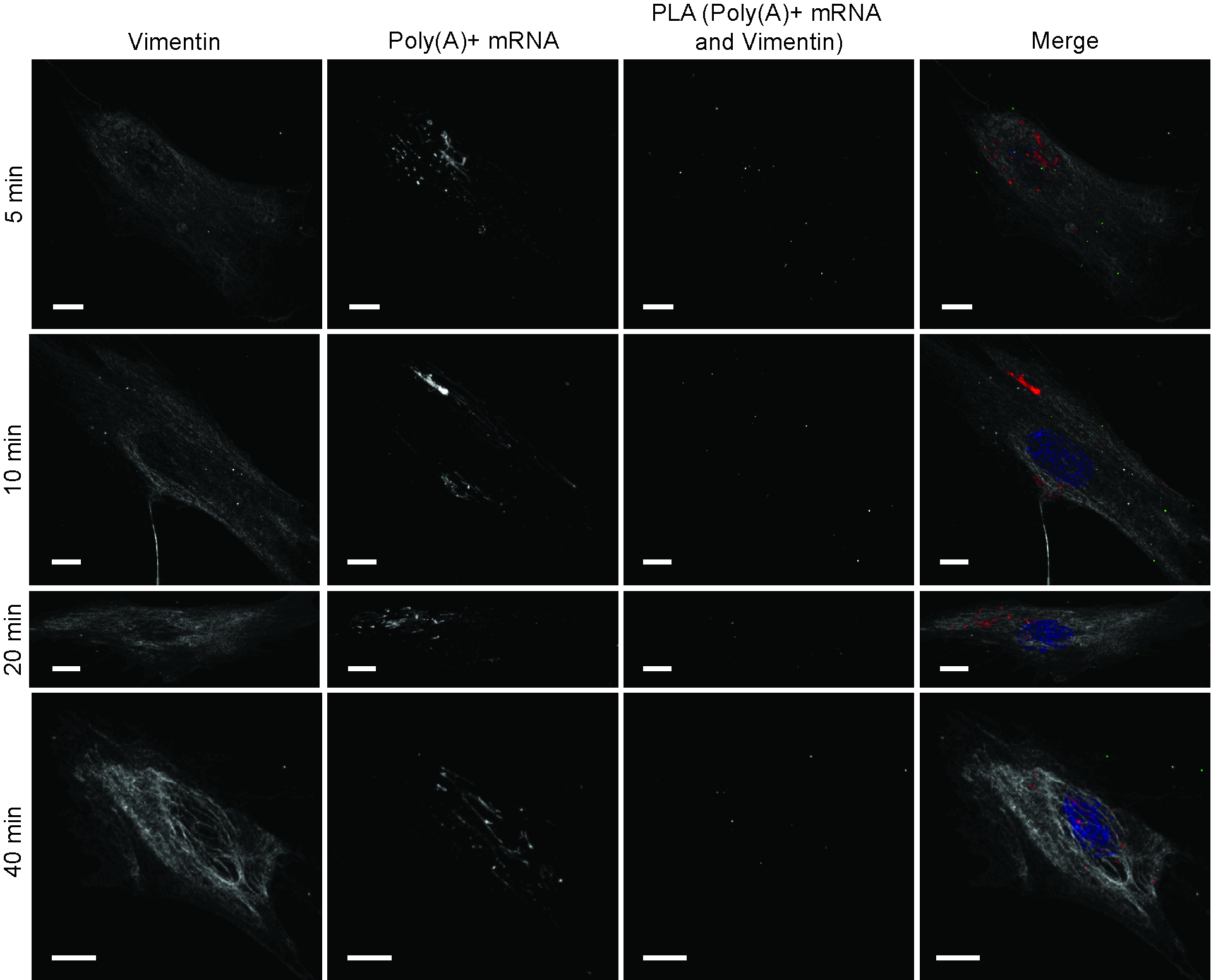

Supplement: Figure S12 — Interactions between poly(A)+ mRNA and vimentin at 5, 10, 20, and 40 min of arsenite exposure. Vimentin IF, poly(A)+ mRNA, and PLA between poly(A)+ mRNA and vimentin were imaged with a laser-scanning confocal microscope. Merged images of vimentin (white), poly(A)+ mRNA (red), PLA (green), and nuclei (blue) are shown. All image planes are represented. Scale bar, 10µm. (TIF) [file pone.0074598.s012.tif]

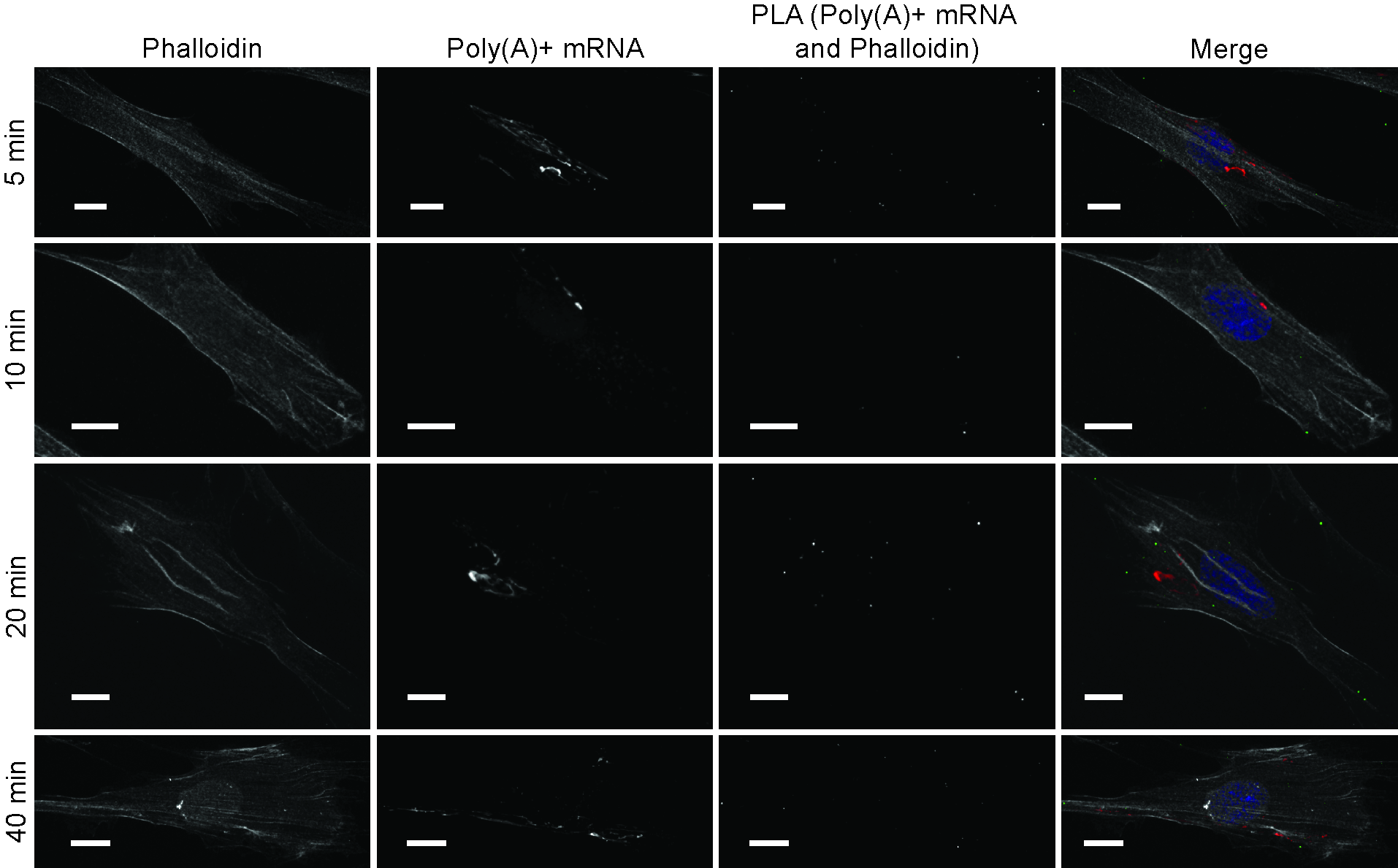

Supplement: Figure S13 — Interactions between poly(A)+ mRNA and phalloidin at 5, 10, 20, and 40 min of arsenite exposure. Phalloidin IF, poly(A)+ mRNA, and PLA between poly(A)+ mRNA and Phalloidin were imaged with a laser-scanning confocal microscope. Merged images of phalloidin (white), poly(A)+ mRNA (red), PLA (green), and nuclei (blue) are shown. All image planes are represented. Scale bar, 10µm. (TIF) [file pone.0074598.s013.tif]

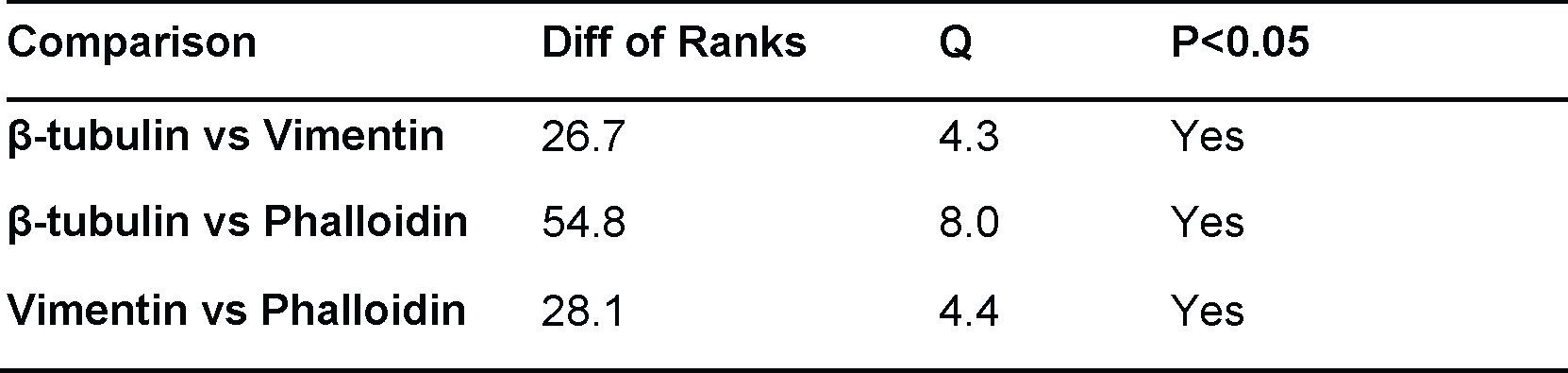

Supplement: Table S1 — Comparisons of the mean percentage of FMTRIP colocalized with PLA (PLA-FMTRIP) in human dermal fibroblasts (HDF) for the interactions between poly(A)+ mRNA and β-tubulin (n=25, mean=2.3%, SD=1.5%), vimentin (n=33, mean=11.5%, SD=9.7%), or phalloidin (n=23, mean=53.7%, SD=19.9%) using Kruskal-Wallis one-way ANOVA on ranks with Dunn’s method for multiple comparison (p < 0.001). (TIF) [file pone.0074598.s014.tif]

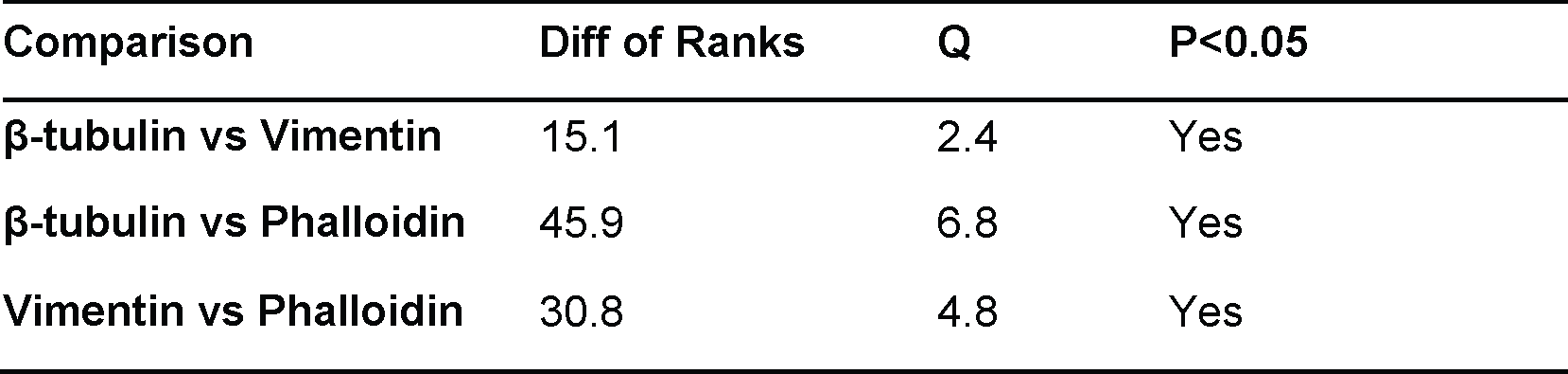

Supplement: Table S2 — Comparisons of the mean PLA frequency in HDF for the interactions between poly(A)+ mRNA and β-tubulin (n=25, mean=0.010µm-3, s.d.=0.007µm-3), vimentin (n=33, mean=0.019µm-3, s.d.=0.016µm-3), or phalloidin (n=23, mean=0.069µm-3, s.d.=0.039µm-3) using Kruskal-Wallis one-way ANOVA on ranks with Dunn’s method for multiple comparison (p < 0.001). (TIF) [file pone.0074598.s015.tif]

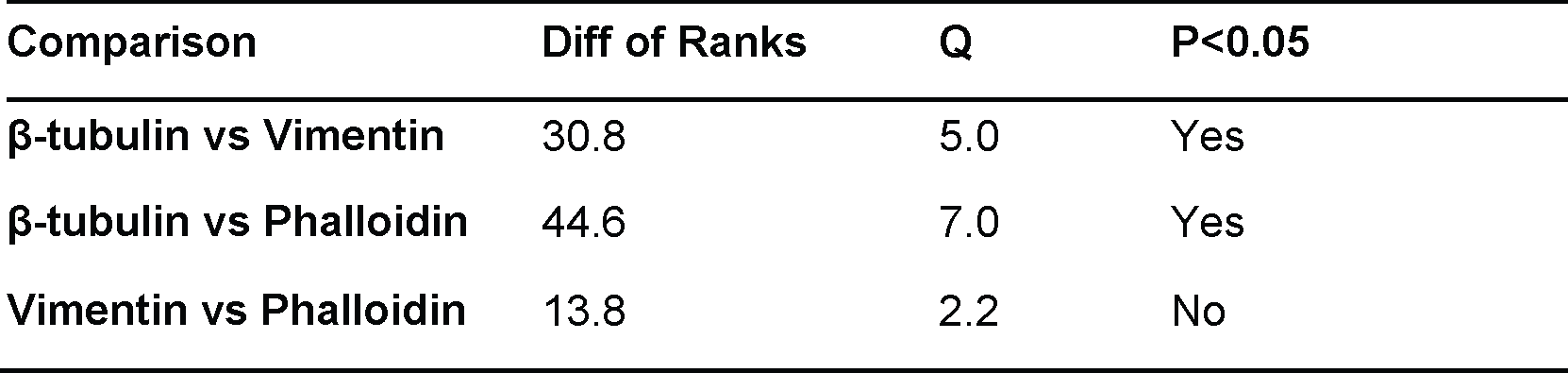

Supplement: Table S3 — Comparisons of the mean percentage of FMTRIP colocalized with PLA (PLA-FMTRIP) in A549 cells for the interactions between poly(A)+ mRNA and β-tubulin (n=26, mean=2.1%, s.d.=1.4%), vimentin (n=28, mean=10.5%, s.d.=6.1%), or phalloidin (n=24, mean=22.9%, s.d.=18.6%) using Kruskal-Wallis one-way ANOVA on ranks with Dunn’s method for multiple comparison (p < 0.001). (TIF) [file pone.0074598.s016.tif]

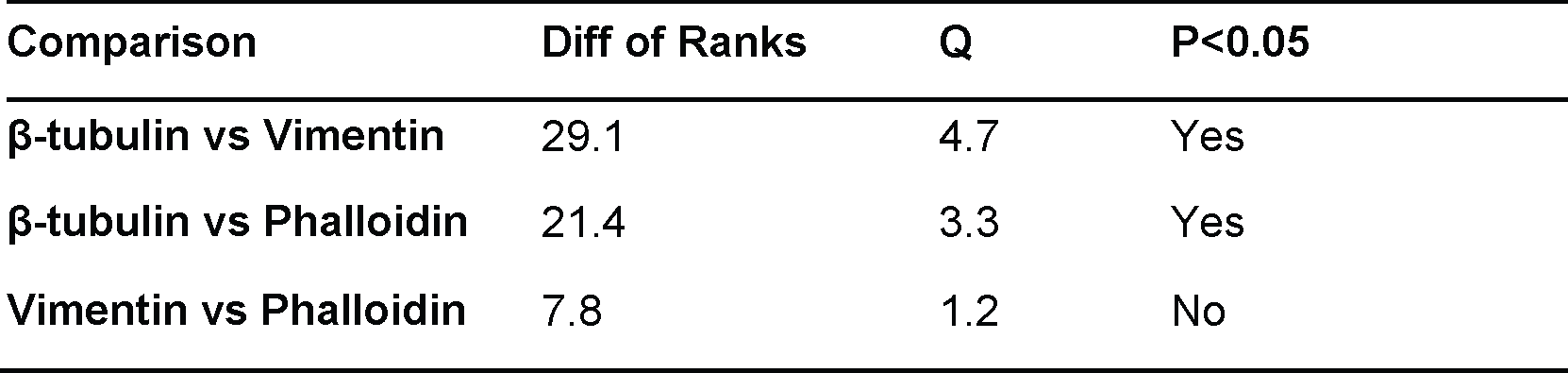

Supplement: Table S4 — Comparisons of the mean PLA frequency in A549 cells for the interactions between poly(A)+ mRNA and β-tubulin (n=26, mean=0.063µm-3, s.d.=0.023µm-3), vimentin (n=28, mean=0.15µm-3, s.d.=0.07µm-3), or phalloidin (n=24, mean=0.13µm-3, s.d.=0.09µm-3) using Kruskal-Wallis one-way ANOVA on ranks with Dunn’s method for multiple comparison (p < 0.001). (TIF) [file pone.0074598.s017.tif]

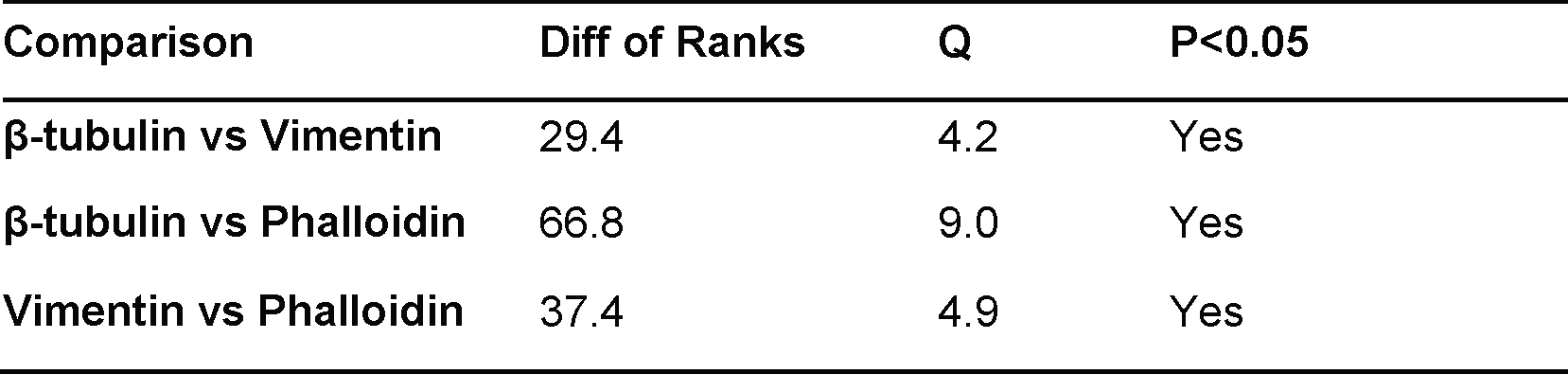

Supplement: Table S5 — Comparisons of the mean percentage of FMTRIP colocalized with PLA (PLA-FMTRIP) in HDF for the interactions between β-actin mRNA and β-tubulin (n=40, mean=3.9%, s.d.=3.2%), vimentin (n=36, mean=12.7%, s.d.=7.9%), or phalloidin (n=30, mean=71.5%, s.d.=20.1%) using Kruskal-Wallis one-way ANOVA on ranks with Dunn’s method for multiple comparison (p < 0.001). (TIF) [file pone.0074598.s018.tif]

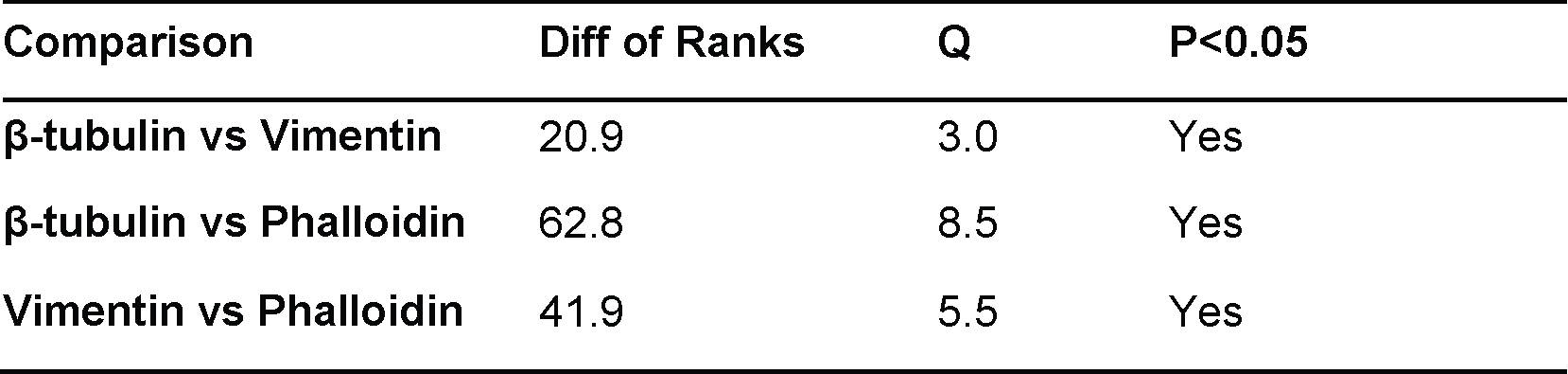

Supplement: Table S6 — Comparisons of the mean PLA frequency in HDF for the interactions between β-actin mRNA and β-tubulin (n=40, mean=0.012µm-3, s.d.=0.011µm-3), vimentin (n=36, mean=0.027µm-3, s.d.=0.023µm-3), or phalloidin (n=30, mean=0.33µm-3, s.d.=0.17µm-3) using Kruskal -Wallis one-way ANOVA on ranks with Dunn’s method for multiple comparison (p < 0.001). (TIF) [file pone.0074598.s019.tif]

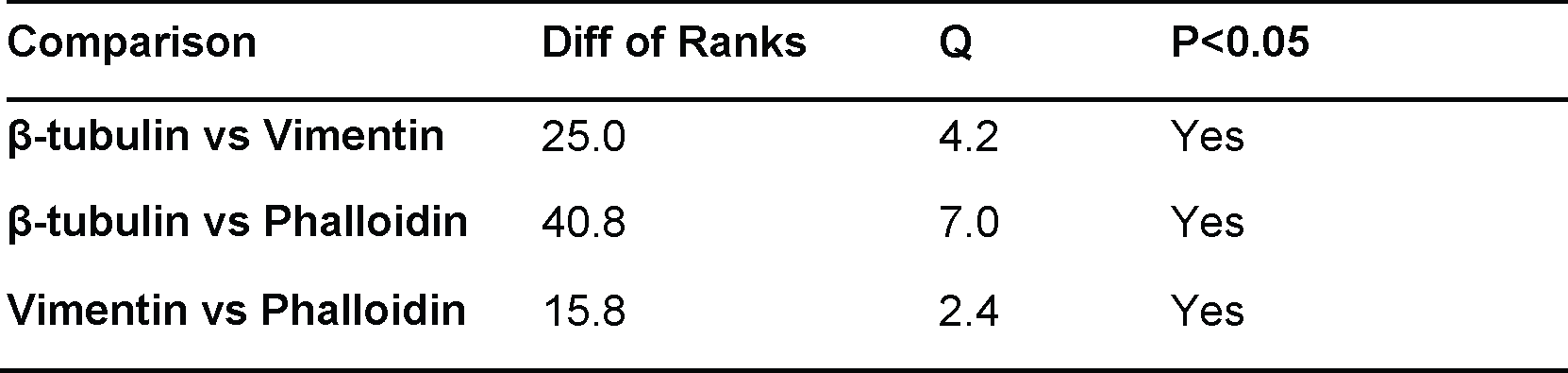

Supplement: Table S7 — Comparisons of the mean percentage of FMTRIP colocalized with PLA (PLA-FMTRIP) in A549 cells for the interactions between β-actin mRNA and β-tubulin (n=31, mean=2.6%, s.d.=1.7%), vimentin (n=19, mean=11.8%, s.d.=5.7%), or phalloidin (n=20, mean=31.3%, s.d.=19.6%) using Kruskal-Wallis one-way ANOVA on ranks with Dunn’s method for multiple comparison (p < 0.001). (TIF) [file pone.0074598.s020.tif]

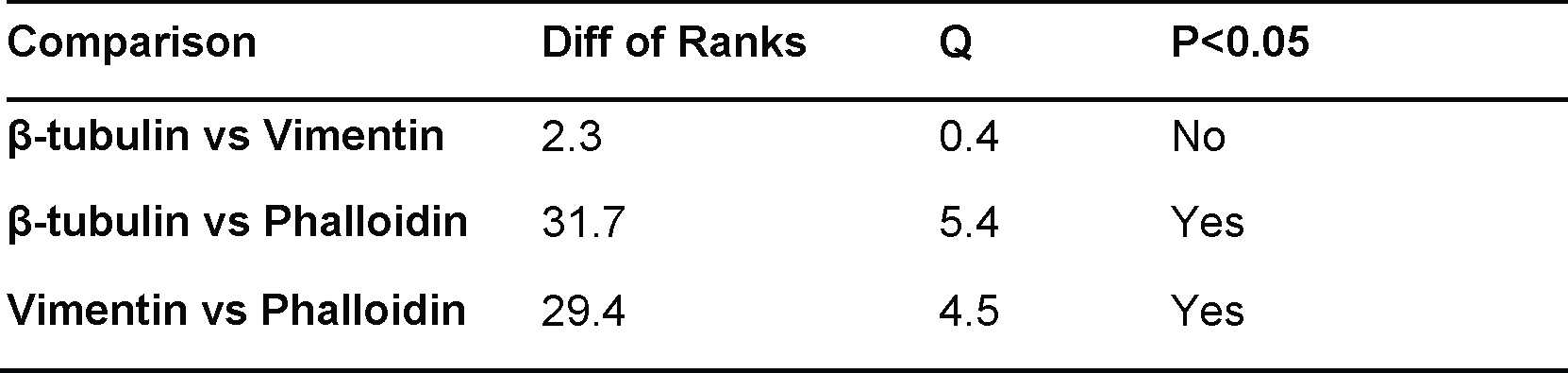

Supplement: Table S8 — Comparisons of the mean PLA frequency in A549 cells for the interactions between β-actin mRNA and β-tubulin (n=31, mean=0.030µm-3, s.d.=0.016µm-3), vimentin (n=19, mean=0.04µm-3, s.d.=0.03µm-3), or phalloidin (n=20, mean=0.14µm-3, s.d.=0.08µm-3) using Kruskal-Wallis one-way ANOVA on ranks with Dunn’s method for multiple comparison (p < 0.001). (TIF) [file pone.0074598.s021.tif]

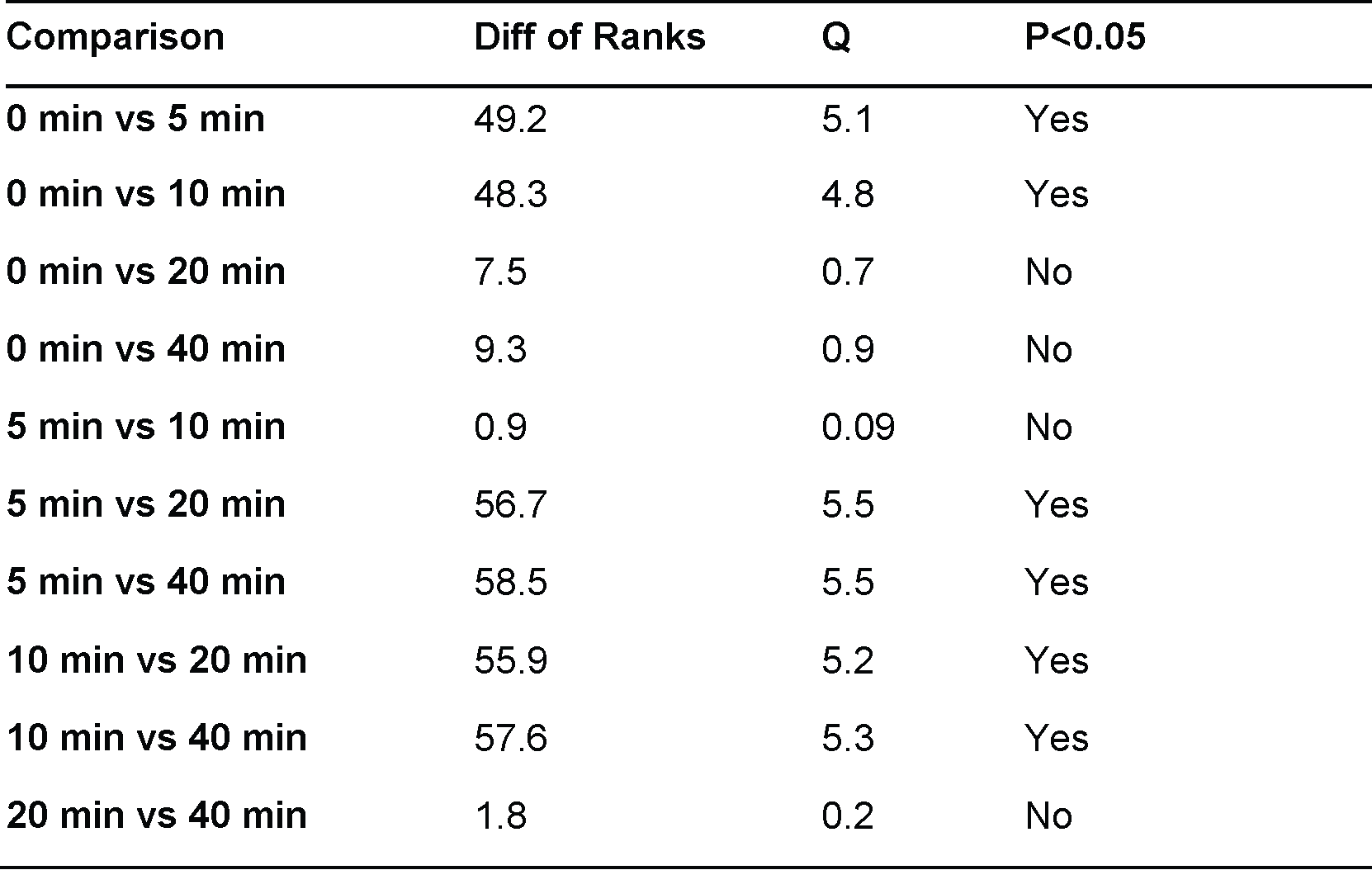

Supplement: Table S9 — Comparisons of the mean percentage of FMTRIP colocalized with PLA (PLA-FMTRIP) for poly(A) + mRNA interactions with β-tubulin in HDF cells exposed to arsenite for 0 (n=28, mean=2.5%, s.d.=1.6%), 5 (n=31, mean=12.8%,s.d.=14.6%), 10 (n=26, mean=11.3%, s.d.=9.1%), 20 (n=22, mean=2.6%, s.d.=3.8%), and 40 min (n=20, mean=2.2%, s.d.=3.0%) using Kruskal-Wallis one-way ANOVA on ranks with Dunn’s method for multiple comparison (p<0.001). (TIF) [file pone.0074598.s022.tif]

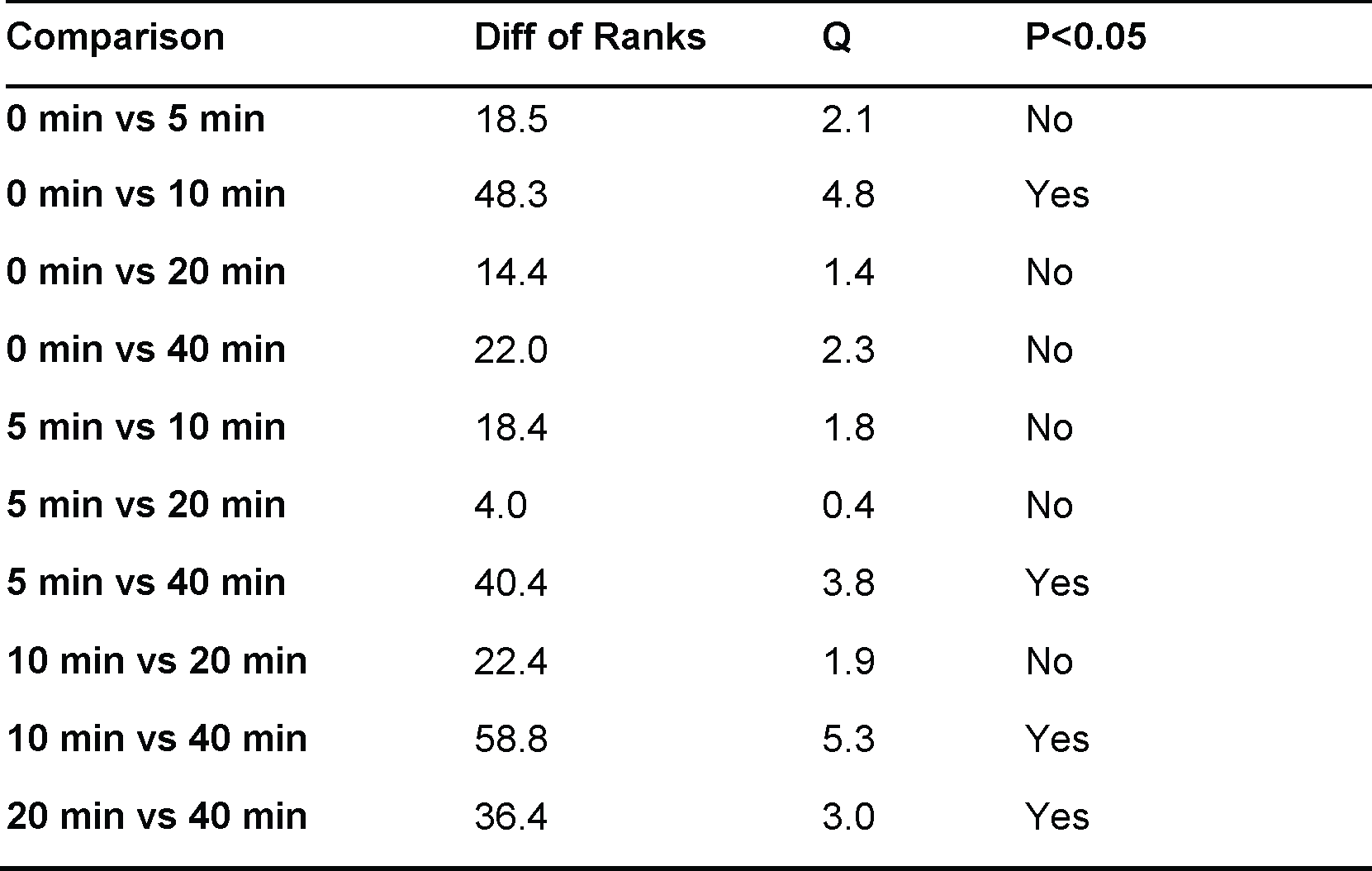

Supplement: Table S10 — Comparisons of the mean percentage of FMTRIP colocalized with PLA (PLA-FMTRIP) for poly(A) + mRNA interactions with vimentin in HDF cells exposed to arsenite for 0 (n=42, mean=11.1%, s.d.=8.8%), 5 (n=23, mean=13.9%,s.d.=6.5%), 10 (n=19, mean=18.7%, s.d.=7.5%), 20 (n=13, mean=13.3%, s.d.=7.1%), and 40 min (n=17, mean=5.9%, s.d.=4.4%) using Kruskal-Wallis one-way ANOVA on ranks with Dunn’s method for multiple comparison (p<0.001). (TIF) [file pone.0074598.s023.tif]

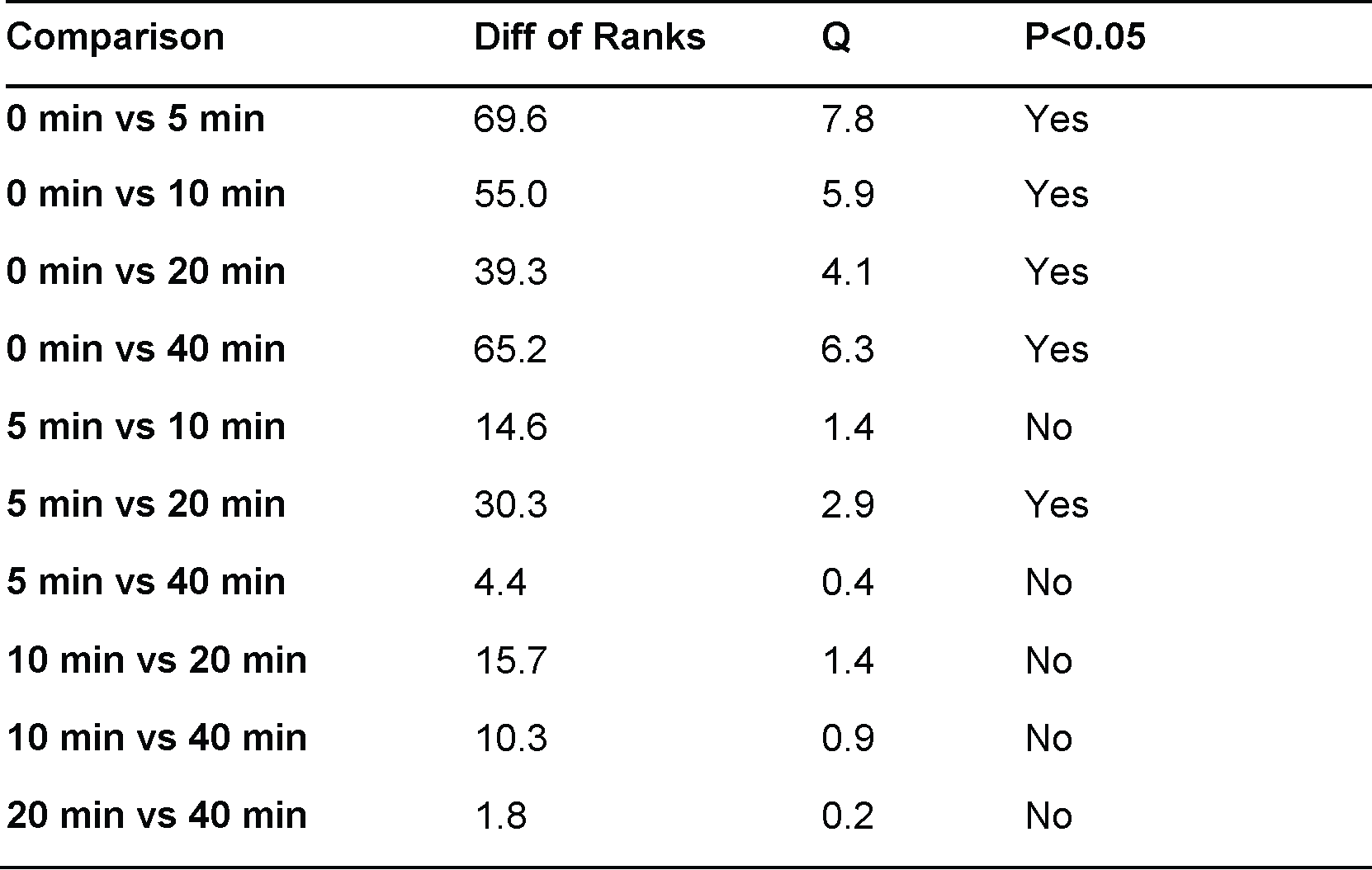

Supplement: Table S11 — Comparisons of the mean percentage of FMTRIP colocalized with PLA (PLA-FMTRIP) for poly(A) + mRNA interactions with F-actin in HDF cells exposed to arsenite for 0 (n=38, mean=49.3%, s.d.=19.4%), 5 (n=25, mean=6.0%,s.d.=5.2%), 10 (n=21, mean=9.6%, s.d.=6.4%), 20 (n=19, mean=15.4%, s.d.=9.4%), and 40 min (n=16, mean=6.8%, s.d.=4.4%) using Kruskal-Wallis one-way ANOVA on ranks with Dunn’s method for multiple comparison (p<0.001). (TIF) [file pone.0074598.s024.tif]

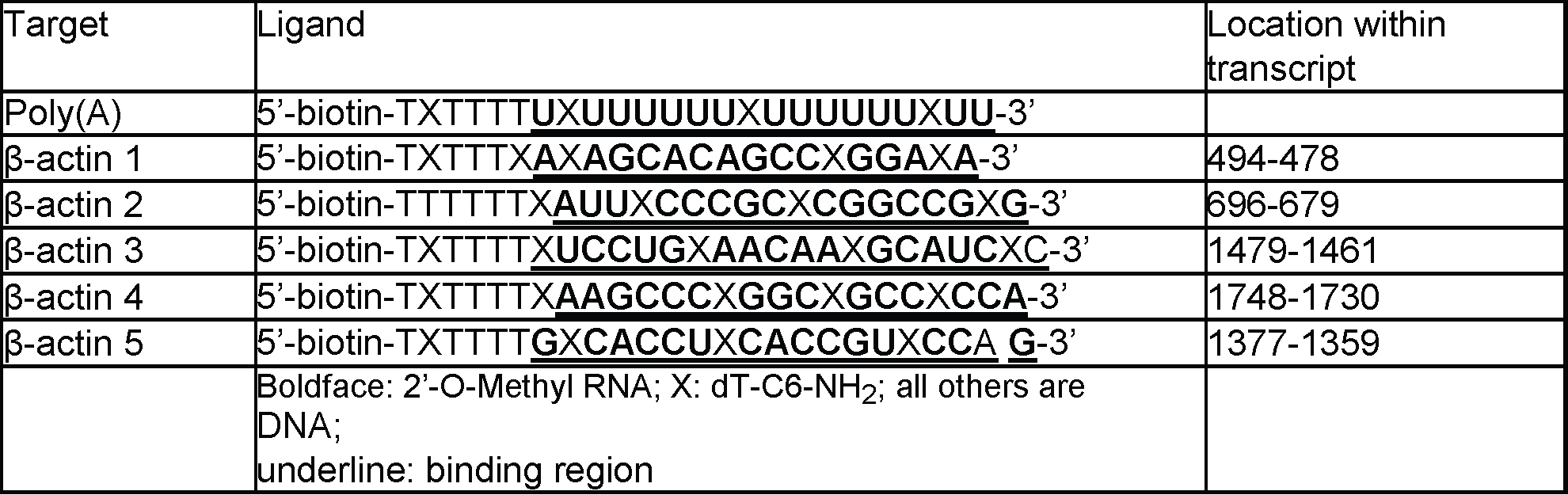

Supplement: Table S12 — Poly(A) + and β-actin mRNA targeting probe sequences and modifications. (TIF) [file pone.0074598.s025.tif]
